# A single gp120 residue can affect HIV-1 tropism in macaques

**DOI:** 10.1371/journal.ppat.1006572

**Published:** 2017-09-25

**Authors:** Gregory Q. Del Prete, Brandon F. Keele, Jeannine Fode, Keyur Thummar, Adrienne E. Swanstrom, Anthony Rodriguez, Alice Raymond, Jacob D. Estes, Celia C. LaBranche, David C. Montefiori, Vineet N. KewalRamani, Jeffrey D. Lifson, Paul D. Bieniasz, Theodora Hatziioannou

**Affiliations:** 1 AIDS and Cancer Virus Program, Leidos Biomedical Research, Inc., Frederick National Laboratory for Cancer Research, Frederick, MD, United States of America; 2 Laboratory of Retrovirology, The Rockefeller University, New York, NY, United States of America; 3 Department of Surgery, Duke University Medical Center, Durham, NC, United States of America; 4 Center for Cancer Research, National Cancer Institute, Frederick, MD, United States of America; 5 Howard Hughes Medical Institute, The Rockefeller University, New York, NY, United States of America; University of Zurich, SWITZERLAND

## Abstract

Species-dependent variation in proteins that aid or limit virus replication determines the ability of lentiviruses to jump between host species. Identifying and overcoming these differences facilitates the development of animal models for HIV-1, including models based on chimeric SIVs that express HIV-1 envelope (Env) glycoproteins, (SHIVs) and simian-tropic HIV-1 (stHIV) strains. Here, we demonstrate that the inherently poor ability of most HIV-1 Env proteins to use macaque CD4 as a receptor is improved during adaptation by virus passage in macaques. We identify a single amino acid, A281, in HIV-1 Env that consistently changes during adaptation in macaques and affects the ability of HIV-1 Env to use macaque CD4. Importantly, mutations at A281 do not markedly affect HIV-1 Env neutralization properties. Our findings should facilitate the design of HIV-1 Env proteins for use in non-human primate models and thus expedite the development of clinically relevant reagents for testing interventions against HIV-1.

## Author summary

Understanding the interactions between viruses and their hosts allows manipulation of primate lentiviruses and the generation of better animal models for HIV/AIDS. Species-dependent differences in cellular proteins that play key roles in virus replication, such as the primary HIV-1 receptor CD4, can limit virus tropism. Our data reveal how adaptation in macaques improves the ability of HIV-1 envelope glycoproteins to use macaque CD4. Moreover, we identify a single amino acid in the HIV-1 envelope glycoprotein CD4 binding site that improves macaque CD4 use by most HIV-1 envelope proteins tested and allows viruses expressing these proteins to replicate efficiently in macaque cells without compromising their sensitivity to various antibodies. These findings should facilitate the development and preclinical evaluation of HIV-1 Env directed prophylactic and therapeutic interventions.

## Introduction

Adaptation of primate lentiviruses to new hosts requires the acquisition of both resistance to species-specific inhibitors and ability to optimally use key host factors critical for virus replication [1]. Understanding how host protein variation drives lentivirus adaptation provides important insights into the evolutionary history of lentiviruses and it can guide efforts to generate improved animal models for AIDS by expanding primate lentivirus host range.

Non-human primates are commonly used to model human HIV-1 infection. Rhesus and pigtail macaques (*Macaca mulatta* and *Macaca nemestrina*, respectively) are not natural hosts for primate lentiviruses and they can develop AIDS-like disease when infected with certain simian immunodeficiency viruses (SIV) derived from the SIV_SM_/SIV_MAC_ lineage. Moreover, chimeric SIV_MAC_-based viruses that encode HIV-1 envelope (Env) glycoproteins allow the evaluation of interventions directed against the HIV-1 Env protein rather than its genetically and antigenically distinct SIV_MAC_ counterpart (reviewed in [1]). For example, approaches that aim to elicit broadly neutralizing antibodies (bnabs) [2–5] by vaccination, or to use bnabs and antibody-like molecules as treatment and prevention agents depend on the use of SHIV models for *in vivo* evaluation [6–15].

SHIV development has been difficult, and the paucity of available SHIVs with robust transmissibility and consistent pathogenicity fails to represent the clinically relevant diversity of circulating HIV-1 strains. Recent efforts have focused on the development of SHIVs incorporating Env proteins derived from transmitted/founder (T/F) HIV-1, viruses that have successfully established initial infections in humans [16, 17]. Compared to most SHIVs used previously, such reagents might represent more relevant targets at which prophylactic and treatment interventions would be aimed [18–21]. Our own studies have described the generation and *in vivo* selection of SHIVs encoding clade B T/F HIV-1 Env proteins, designated SHIV_1054_ and SHIV_AD081_, that are pathogenic in rhesus macaques and lead to AIDS-like disease in a subset of infected rhesus macaques without requiring animal-to-animal passage [22]. Nevertheless, pathogenicity of these SHIVs is not consistent. Indeed, most SHIVs, have not given persistent high level viral replication, authentic HIV-like pathogenesis and progression to AIDS in macaques. Derivation of pathogenic isolates from T/F-SHIVs has required animal-to-animal passage [18–21].

In addition to SHIVs, animal models based on HIV-1 strains, rather than SIV, are also being developed by generating minimally modified simian-tropic HIV-1 strains, designated stHIVs, that can replicate in pigtail macaques [23, 24]. Recently, by employing serial animal-to animal passage, we derived an stHIV that can consistently cause AIDS-like disease in pigtail macaques subjected to transient CD8+ cell depletion during acute infection [25]. Although restriction factor evasion was critical for the development of stHIV, adaptation of HIV-1 Env to pigtail macaques also likely played an important role in the development of this model, similar to HIV-1 Env adaptation in rhesus macaques in the context of SHIVs.

The goal of the present study was to identify properties of HIV-1 Env proteins that determine their ability to support virus replication in macaques and whether these properties can be exploited to guide development of additional SHIV or stHIV strains. Our experiments show that most HIV-1 Env proteins have reduced ability to use macaque CD4 compared to human CD4, in agreement with other published studies [21, 26] and further demonstrate that selection by SHIV or stHIV by adaptation in macaques can improve this ability. Notably, we identify a single amino-acid change, A281T/V, within the CD4 binding site that improves the ability of HIV-1 Env proteins to use macaque CD4 and enables SHIV replication in macaque T-cells. Although previous studies have uncovered other individual residues in HIV-1 Env proteins that can improve macaque CD4 use [21, 27, 28], only the A281T/V change identified herein occurs repeatedly during HIV-1 Env adaptation in macaques. Importantly, unlike some of the other mutations, the A281T/V does not have major effects on Env protein neutralization properties. These findings identify a host range determinant in HIV-1 and should facilitate the expansion of the clinically relevant viral repertoire for use in animal models.

## Results

### Adaptation of SHIV_1054_ in rhesus macaques

**Fig 1:**
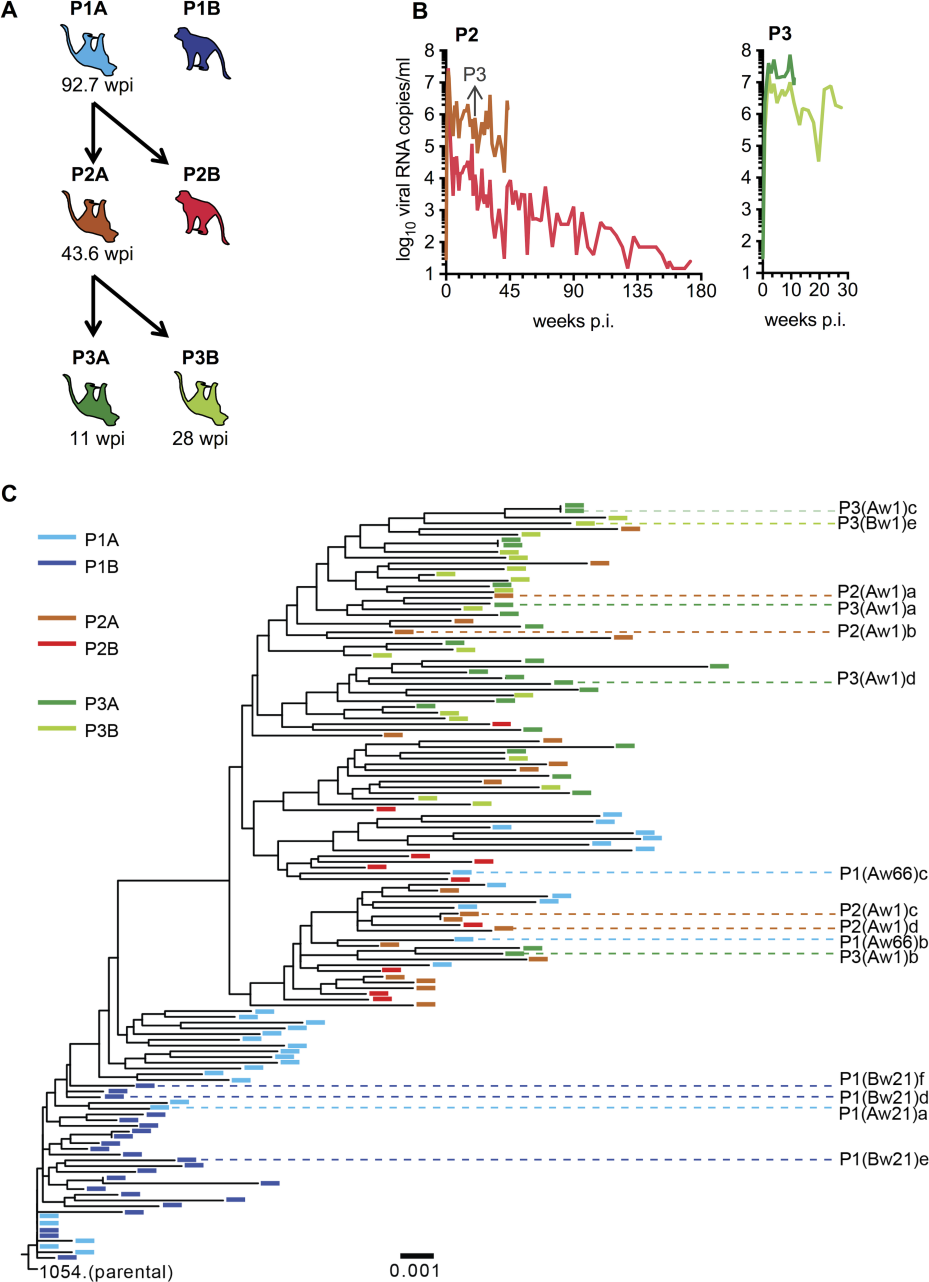
Adaptation of 1054 Env *in vivo*. **(A)** Schematic representation of *in vivo* passaging of SHIV_1054_. Animals indicated by inverted cartoons were euthanized for clinical cause including AIDS defining symptoms and the time of euthanasia (weeks post-inoculation) is indicated under each animal. Colors indicate passage and animal designation A/B and the same color scheme is maintained throughout the figures. (**B)** Plasma viremia in SHIV_1054_ infected rhesus macaques. Grey arrow indicates the time point at which blood was taken to initiate the subsequent passage. (**C)** Phylogenetic analysis of Env sequences isolated from plasma samples of macaques infected with SHIV_1054_ and subsequent animal passages outlined in (A). Clones analyzed in this study are highlighted.

We previously demonstrated that *in vivo* competition following inoculation of macaques with pools of viruses allows the identification of unadapted HIV-1 Env proteins that confer high levels of replication in the context of a SHIV [22]. Although these SHIVs caused AIDS-like disease in some rhesus macaques, progression to disease was inconsistent. We selected one such SHIV, SHIV_1054_, and employed serial animal-to-animal passage to improve its pathogenicity (Fig 1A). Blood obtained at 66 weeks post-inoculation (p.i.) from a SHIV_1054_-infected rhesus macaque, here designated P1A, that eventually succumbed to AIDS [22] was used to inoculate two naïve rhesus macaques, P2A and P2B (Fig 1A). Both P2 macaques developed high levels of acute viremia (Fig 1B) and peripheral CD4+ T cell levels declined markedly in both animals following inoculation, partially recovered in P2A and eventually declined further (S1A Fig). A progressive decline to AIDS defining levels of CD4+ T cells (S1A Fig) occurred in P2A which succumbed to AIDS-like disease at 44 weeks p.i. (Fig 1A and 1B). Blood from P2A obtained at 21 weeks p.i. was used to initiate a third passage of SHIV_1054_ in two macaques and both P3 animals exhibited more rapid progression to disease, requiring euthanasia at weeks 11 and 28 weeks p.i. (Fig 1B). Peripheral CD4+ T cells declined dramatically in both infected animals upon inoculation and did not recover (S1A Fig).

To understand the nature of *in vivo* adaptation that occurred during SHIV_1054_ passage, single-genome sequence analysis of Env-coding sequences was performed using plasma samples obtained at various time points p.i. from all infected animals and, as expected, revealed progressive accumulation of sequence changes (Fig 1C). To assess the potential functional consequences of these changes, representative Env clones derived from P1 macaques at weeks 21 and 66 p.i. and P2 and P3 macaques at week 1 p.i., were amplified by PCR and re-introduced into the parental SHIV backbone (Fig 1C). Adapted Env sequences were named according to passage number and time of blood draw. For example “P2(Aw1)a” would refer to an individual sequence recovered at passage 2, animal A at 1 week post infection, designated clone ‘a’.

**Fig 2:**
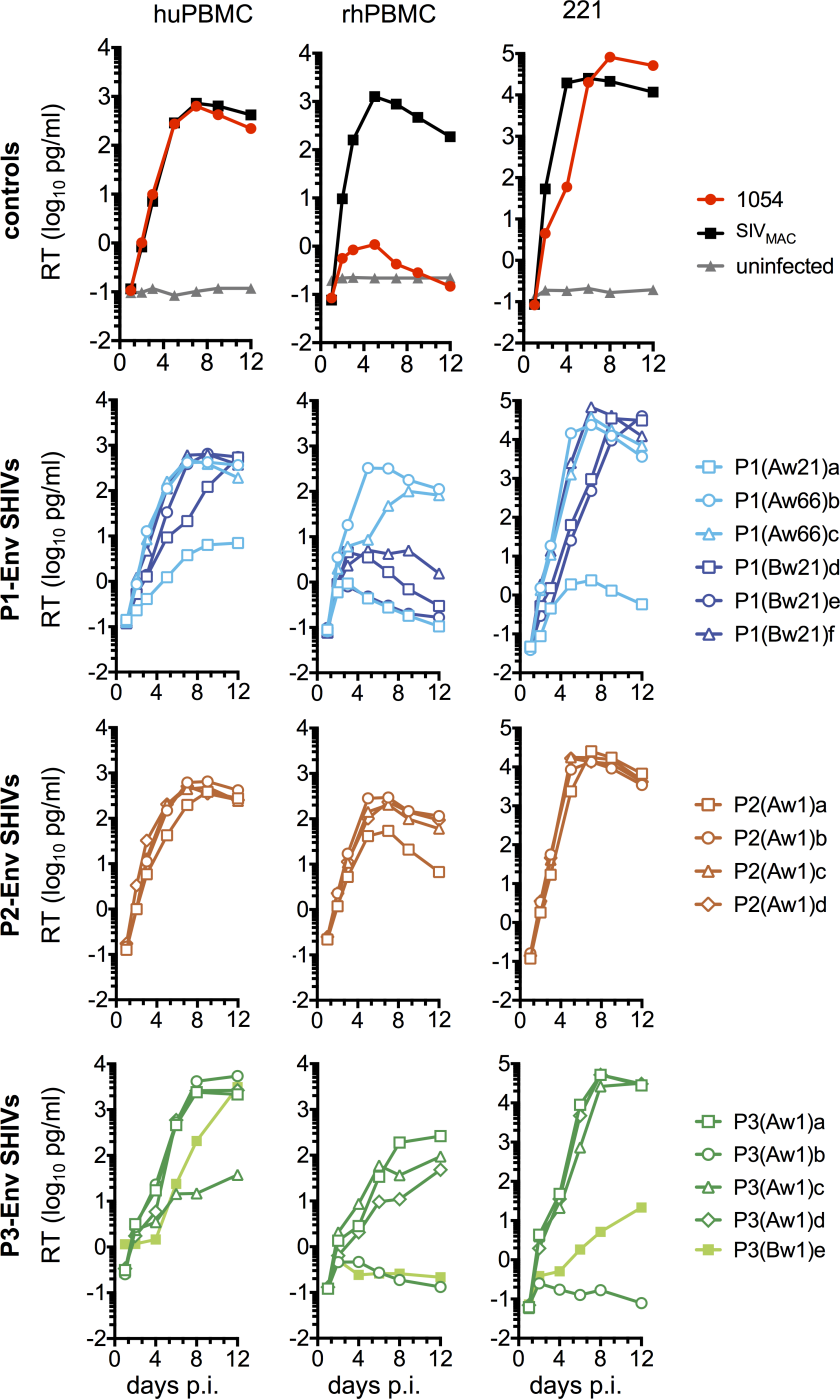
*In vitro* replication of SHIVs expressing adapted 1054 Env proteins. Replication of SHIVs expressing the parental or adapted 1054 Env proteins in huPBMC, rhPBMC and rhesus 221 immortalized T cells. SIV_MAC_ was used as control. Individual SHIV stocks were normalized for RT and 100pg RT of each virus was used per 1x10^5^ activated hu/rhPBMC. For 221 cells 50pg RT of each virus was used per 1x10^5^ cells. Replication was measured by RT in samples collected at the indicated times post-inoculation.

We next determined the ability of the cloned Env sequences derived from macaques to support viral replication *in vitro*. In huPBMC, SHIV_1054_ and derivatives encoding most of the adapted clones replicated efficiently, though for a few adapted clones from P1 and P3 replication was somewhat delayed or attenuated (Fig 2). In contrast, large differences in replication were observed in rhesus macaque cells (Fig 2). In rhPBMC, SHIV_1054_ and three SHIVs expressing adapted Env clones from P1 and P3 barely replicated, however, the majority of SHIVs expressing adapted Env clones replicated markedly more efficiently than the parental SHIV_1054_ (Fig 2). These differences were less pronounced in 221 cells, an immortalized rhesus macaque T-cell line [29]. The parental SHIV_1054_ replicated efficiently is 221 cells as did the majority of SHIVs expressing adapted Env proteins (Fig 2). SHIVs with impaired replication in rhPBMC also exhibited defects in replication in 221 cells to various degrees; replication of SHIV-P1(Aw21)a and SHIV-P3(Aw1)b was extremely low in 221 cells, whereas SHIV-P1(Bw21)d and SHIV-P1(Bw21)e replicated more efficiently in 221 cells than rhPBMC but were nonetheless delayed (peak virus levels reached at 9 and 12 days p.i. respectively) compared to most viruses (peak virus levels reached at day 7 p.i.) (Fig 2). SHIV-P3(Aw1)e also replicated more efficiently in 221 cells compared to rhPBMC but the delay in replication in 221 was more prominent than SHIV-P1(Bw21)d and SHIV- P1(Bw21)e (Fig 2).

We also tested replication of a subset of the SHIVs in CD8 depleted rhesus macaque cells (S1B Fig). Critically, the rank order of replication efficiency for SHIV_1054_ and derivatives mirrored that obtained in 221 cells, demonstrating that this cell line maintains species-specific differences that impact SHIV replication efficiency. However, like CD4 enriched primary T-cells, 221 cells are more permissive than rhPBMC. Altogether, the data showed that, with the exception of a few clones, most adapted Env sequences conferred improved SHIV replication compared to the parental 1054 Env protein, specifically in macaque cells.

### Adaptation of SHIVs in rhesus macaques leads to improved use of macaque CD4

**Fig 3:**
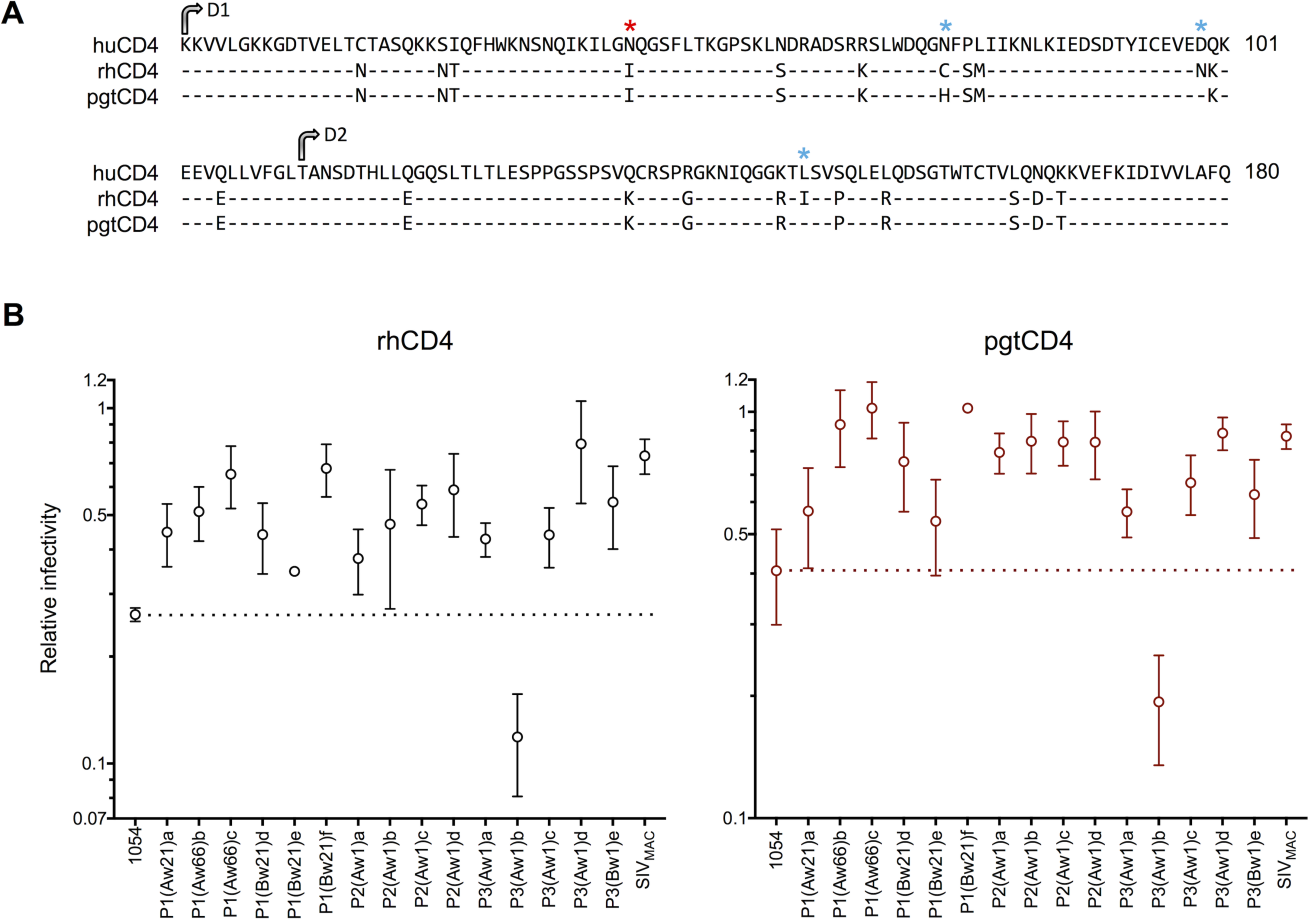
*In vitro* ability of adapted 1054 Env proteins to use macaque CD4. **(A)** Amino acid (aa) alignment of the N-terminal domain of human CD4 and the two major variants of macaque CD4, designated as rh and pgt CD4. Residue 39 shown to be important for function as HIV-1 Env receptor is marked with a red asterisk. Blue asterisks mark polymorphisms between rh and/or pgt CD4 variants. Domains D1 and D2 are indicated with arrows. Numbering starts at the first residue of the D1 domain (aa 25 in the CDS). **(B)** SHIV infectivity on cells expressing human or macaque CD4 variants. SHIVs expressing parental or *in vivo*-adapted 1054 Env proteins were titrated on indicator Helios cells expressing hu/rh/pgtCD4. SIV_MAC_ (SIV_MAC239_) was used for comparison. Infectivity was quantified by measuring luciferase expression in infected cells as Relative Light Units (RLU) and plotted as the ratio of RLU obtained in rh/pgtCD4-Helios over RLU obtained in huCD4-Helios. Average and standard deviation from two independent experiments is shown.

Replication of SHIVs in macaque cells could be potentially limited by the inability of HIV-1 Env proteins to engage macaque CD4. Indeed, a prior study has shown that a single amino-acid (aa) difference at position 39 of macaque CD4 decreases HIV-1 receptor function compared to human CD4 [26]. CD4 is well conserved among individual humans, with only one common nonsynonymous single nucleotide polymorphism (SNP) [30], but analysis of CD4 polymorphism in macaques has been limited, often using few animals or partial sequence analysis [26, 31]. We analyzed CD4 cDNA sequences from 17 rhesus and 14 pigtail macaques and found no less than twelve variants encoding distinct protein sequences (that included all previously reported macaque CD4 sequences). Two different variants were clearly predominant in rhesus macaques and pigtailed macaques (S2A Fig) and were thus designated rhCD4 and pgtCD4 (Fig 3A), though we note that the rhCD4 variant was also found in four heterozygous pigtail macaques. In pigtail macaques, 7/14 animals were homozygous for pgtCD4, one was heterozygous for pgtCD4 and rhCD4 and 6 animals expressed either pgtCD4 or rhCD4 along with one of four minor variants. In rhesus macaques, 7/12 animals were homozygous for rhCD4, while 5 expressed rhCD4 and one minor variant. Of note, variation within individual animals in CD4 has been previously observed in sooty mangabeys and African green monkeys [31]. In macaques, the minor variants differed from rhCD4 and pgtCD4 at one or two amino acid positions, mainly toward the C-terminus of the protein (S2A Fig) and some also had synonymous changes (Genbank accession numbers MF769792-804). All macaque CD4 variants differed from human CD4 at aa 39 (Fig 3A). Although the biological significance of CD4 polymorphism in macaques is unknown and warrants further examination, for the purposes of this study we focused our attention on the two major variants, rhCD4 and pgtCD4, that were represented in all animals.

To determine the ability of these CD4 variants to function as receptors for HIV-1 Env, we generated reporter cell lines, designated Helios cells, based on HeLa cells that express the nanoluciferase gene under the control of the HIV-2 LTR (that responds to both HIV-1 and SIV_MAC_). Subsequently, Helios derivatives were engineered to stably express huCCR5 and either huCD4, rhCD4 or pgtCD4 (S2C Fig). Identical, high levels of CD4 were present in Helios cells expressing each of the CD4 variants (S2B Fig). Viruses were titrated on all cell lines and results expressed as the relative infectivity of each virus on rh/pgt-CD4-Helios compared to huCD4-Helios cells. We note, that 221 cells express a minor variant that differed from the major rhCD4 variant used in Helios at two amino acid positions; I144L and A324T.

SIV_MAC_ infectivity was similar in cells expressing huCD4, rhCD4 or pgtCD4, (ratio approaching 1) but the parental, unadapted SHIV_1054_ was less infectious on rhCD4-Helios and pgt-CD4-Helios compared to huCD4-Helios (Fig 3B). In contrast, the relative infectivity of the majority of adapted SHIV_1054_ Env variants on rhCD4-/pgtCD4-Helios was higher than the parental SHIV_1054_ (Fig 3B), suggesting that *in vivo* adaptation resulted in a more efficient use of macaque CD4 receptor variants. Unlike most SHIVs expressing adapted clones, SHIV-P3(Aw1)b was impaired in its ability to infect rh/pgtCD4-Helios (Fig 3B) correlating with its inability to replicate efficiently in macaque cells (Fig 2). Additionally, the overall infectivity of SHIVs expressing Env proteins P1(Aw21)a, and P3(Bw1)e was low in all Helios cells (S3 Fig and compared with Fig 3B) again correlating with delayed or decreased levels of replication in both human and macaque cells (Fig 2). Despite these exceptions, these data show that, in general, adaptation of HIV-1 Env in macaques improves their ability to use macaque CD4 proteins.

### Identification of Env determinants of improved macaque CD4 use

**Fig 4:**
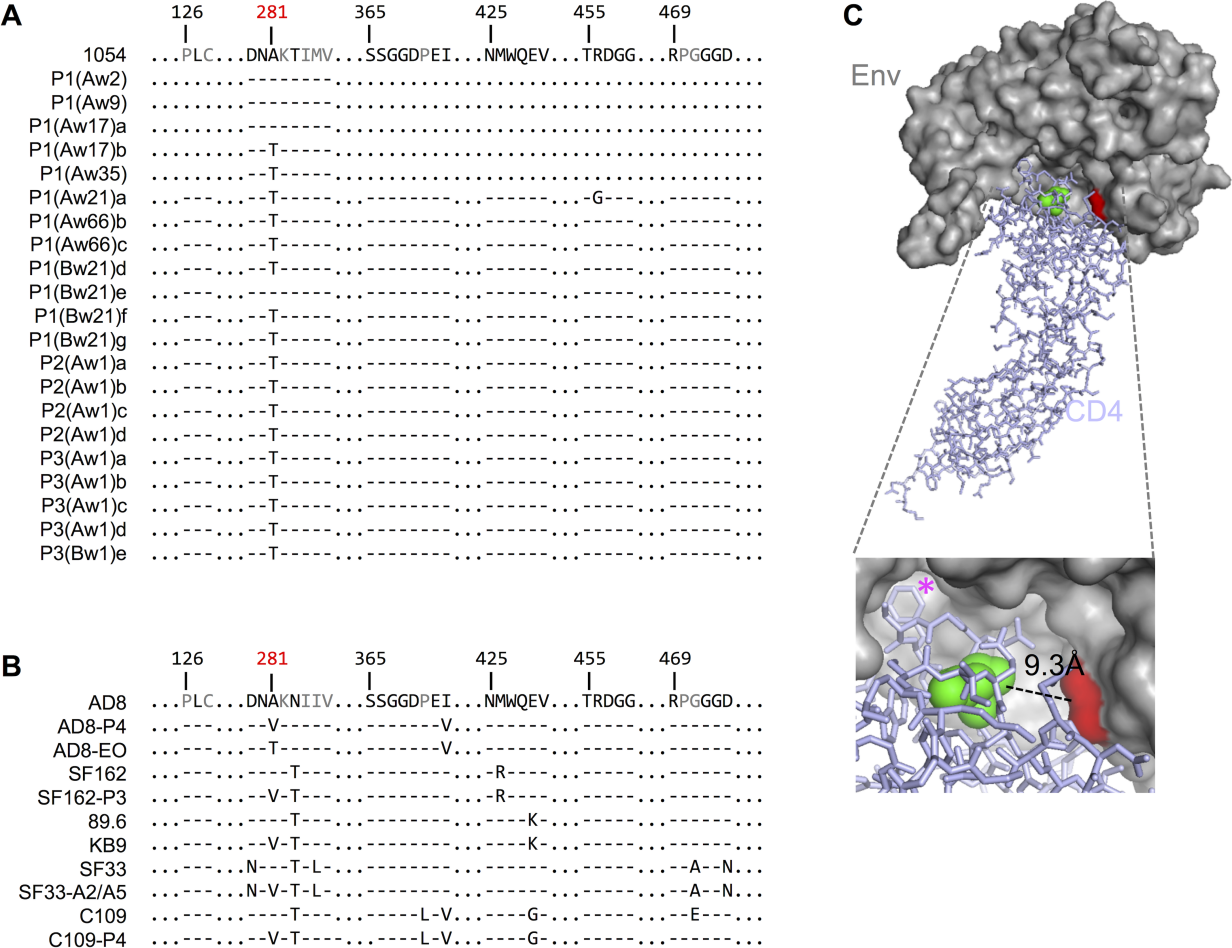
Amino acid changes in the CD4 binding site of gp120. **(A)** Amino acid sequence alignments of the CD4 binding site of HIV-1 gp120 proteins prior to (top) and following passage in macaques of 1054. Clones obtained from animal P1A at weeks 2, 9 and 35 post-inoculation are represented by sequences P1(Aw2), P1(Aw9) and P1A(w35) respectively. Individual clones from animal P1A obtained from week 17 post-inoculation and subsequent weeks and passages are shown. Residues are numbered as in [37] and residues shown to interact directly with CD4 are in black. (B) Amino acid sequence alignments as in (A) of AD8, SF162, 89.6, SF33 and C109 HIV-1 Env aa sequences prior to (top) and following *in vivo* adaptation (bottom). **(C)** Structure of gp120 bound to soluble human CD4 (modified from [37] using PyMol). Surface representation of HIV-1 gp120 is shown in grey, with aa A281 with in red. Stick representation of huCD4 in light purple with residue N39 shown as green spheres. Calculated distance between gp120-A281 and huCD4-N39 is shown. The magenta asterisk marks Phe43 in CD4 (that is conserved between human and macaque proteins) involved in the interaction with gp120.

To identify changes that were responsible for improved macaque CD4 use by adapted Env proteins, we compared the sequences of adapted SHIV_1054_ clones with the parental SHIV_1054_ Env. Although many changes accumulated in the SHIV_1054_ Env coding sequence during *in vivo* passage, only a single change, A281T, in the CD4 binding site was common to the majority of clones (Fig 4A). This change was first detected at week 17 p.i. in animal P1A (Fig 4A). However, due to the limited number of sequences analyzed from the early time points we cannot exclude the possibility that this change appeared earlier. Notably, the A281T change was present in all Env clones sequenced at week 35 p.i. and subsequent time points (Fig 4A).

We compared the *in vivo* adaptation of 1054 Env to the adaptation of a different HIV-1 Env sequence, AD8, that we previously passaged in pigtail macaques in the context of stHIV [25]. We compared the parental AD8 sequence with twenty adapted AD8 Env coding sequences obtained from the first pigtailed macaque animal to succumb to HIV-1-induced AIDS (at passage 4 (P4)). Although multiple mutations had accumulated in individual clones, all had a change at position A281: 11 clones had A281T, 8 clones had A281V and 1 clone had A281I. Env coding sequences from clones obtained from subsequent passages (P5 and P6) almost exclusively contained V at position 281 [25]. The A281 mutation was the only change in the CD4-binding site common to all our pigtail macaque-adapted AD8 sequences, (represented here by a functional Env clone AD8-P4 (Fig 4B)), as well as the adapted 1054 Env clones (Fig 4A). Notably, an A281T mutation also occurred in an independent adaptation of the AD8 Env in rhesus macaques in the context of a SHIV, [32, 33], represented by AD8-EO (Fig 4B).

Given that A281T/V mutations occurred in all three independent adaptation experiments, we inspected additional published sequences and found that an A281V change also occurred during the macaque adaptation of several other SHIVs, specifically SHIV_SF162P3_ [34], SHIV_KB9_ (derived from SHIV_89.6_) [35], SHIV_SF33A2_ [36] and clade C SHIV_C109P4_ ([18] and Cecilia Cheng-Mayer personal communication) (Fig 4B). (SIV divergence in this region precluded identification of the corresponding residue in SIV Env proteins.)

These findings suggested a role for residue 281 in the adaptation of HIV-1 Env proteins in macaques. Inspection of the monomeric HIV-1 gp120-huCD4 crystal structure [37] revealed that A281 is positioned at the gp120-CD4 interface (Fig 4C). Remarkably, this position in HIV-1 gp120 is proximal to CD4 aa 39 that confers poor HIV-1 receptor function on macaque CD4 proteins [26]. Therefore, variants at position 281 are optimally positioned to directly and specifically affect the interaction between HIV-1 Env and macaque CD4.

### A role for HIV-1 Env amino acid 281 in species tropism

**Fig 5:**
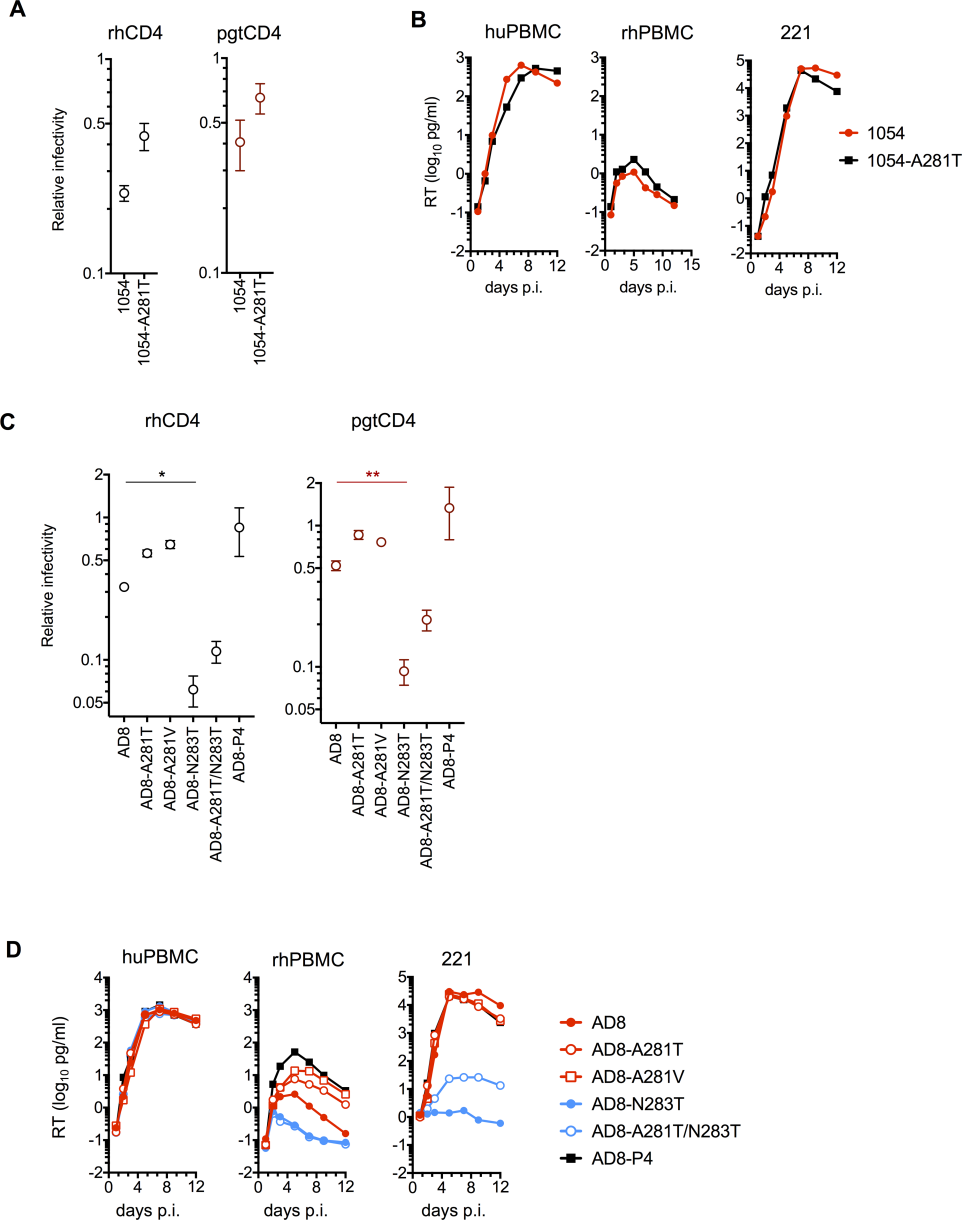
Role of aa 281 in HIV-1 gp120 use of macaque CD4. **(A)** SHIV infectivity on cells expressing human or macaque CD4 variants. SHIVs expressing parental or A281T mutant 1054 Env proteins were titrated on indicator Helios cells expressing hu/rh/pgtCD4. Infectivity was quantified by measuring luciferase expression in infected cells as Relative Light Units (RLU) and plotted as the ratio of RLU obtained in rh/pgtCD4-Helios over RLU obtained in huCD4Helios. Average and standard deviation from two independent experiments is shown. (**B)** Replication of SHIVs expressing the parental or mutant 1054 Env proteins in huPBMC, rhPBMC and rhesus 221 immortalized T cells. SHIV stocks were normalized for RT and 100pg RT of each virus was used per 1x10^5^ activated hu/rhPBMC. For 221 cells 50pg RT of each virus was used per 1x10^5^ cells. Replication was measured by RT in samples collected at the indicated times post-inoculation. **(C)** Infectivity of SHIVs expressing parental or mutant AD8 Env proteins as in (A). The differences between AD8 and AD8-N283T reached statistical significance *P = 0.02 and **P = 0.004. **(D)** Replication of SHIVs expressing the parental or mutant AD8 Env proteins as in (B).

To determine the effects of mutations at residue A281 on the ability to use macaque CD4, we introduced A281T into the parental SHIV_1054_. Unlike most HIV-1 Env proteins, the parental SHIV_1054_ was able to use macaque CD4 variants with modest efficiency (~2.5–4 fold less efficiently than huCD4). Introduction of the A281T change further improved this ability (Fig 5A) to a degree comparable to the *in vivo* adapted SHIV_1054_–derived Env proteins (Fig 3B). As SHIV_1054_ replicated efficiently in huPBMC and 221 cells the A281T mutation did not appreciably impact this ability, however, in rhPBMC where SHIV_1054_ is attenuated, the A281T change caused a small increase in replication efficiency (Fig 5B).

To determine the effects of the A281 change in the context of the AD8 Env, we generated SHIVs expressing the parental AD8 Env and introduced the A281T or V mutations therein. We also generated a SHIV expressing the adapted AD8 Env from our passage 4 stHIV clone, AD8-P4. As was the case with SHIV_1054_, SHIV_AD8_ infectivity on rh/pgtCD4-Helios cells was reduced only modestly (~3-fold) compared to huCD4-Helios (Fig 5C). Nevertheless, introduction of the single amino acid change at position A281 further decreased these differences (Fig 5C). Notably, the effects of the A281T/V mutations on use of macaque CD4 were comparable to that of *in vivo* adaptation in AD8-P4. Although these effects were modest, we note that AD8, like 1054, is unusual amongst HIV-1 Env proteins in that it can use macaque CD4 variants quite efficiently (see below). Although the A281 mutations in AD8 Env did not affect replication in huPBMC or 221 cells, they noticeably improved replication in rhPBMC (Fig 5D).

Like 221 cells [29], human and macaque PBMC, Helios cells express high levels of CD4 (S2B and S2C Fig). However, CD4 density on macrophages in humans is relatively low [38] and consequently macrophage-tropic viruses have adapted to low levels of CD4 [38–40]. Interestingly, AD8 is a macrophage tropic Env and maintains macrophage tropism in pigtail macaques [23]. We reasoned that the ability to use to low levels of CD4, as is the case for macrophage tropic HIV-1 Env proteins, could enhance their ability to use CD4 molecules that are normally not used efficiently, such as macaque CD4. Therefore, we introduced the N283T mutation into our SHIV_AD8_, a change known to specifically disrupt the ability of AD8 Env to infect macrophages and render it T-cell tropic [41]. The N283T mutation diminished SHIV_AD8_ infectivity on Helios cells expressing rhCD4 or pgtCD4 by ~5- to 10- fold but had no effect in cells expressing huCD4 (Fig 5C). Consequently, while all SHIV_AD8_ derivatives replicated equivalently in huPBMC, the N283T mutation abolished replication specifically in 221 and rhPBMC (Fig 5D). Notably, the A281T mutation was able to partially restore the infectivity of AD8-N283T on rhCD4 or pgtCD4 variants and enabled low levels of replication in 221 cells (Fig 5C and 5D). The effects of the A281T mutations on AD8-N283T were not sufficient to improve replication in rhPBMC.

Taken together these data demonstrate a correlation between the ability of SHIVs to use macaque CD4 (a property influenced by Env residue 281) and replication efficiency in rhesus macaque cells *in vitro*.

### Role of residue 281 in macaque CD4 use in the context of divergent HIV-1 Env proteins

**Fig 6:**
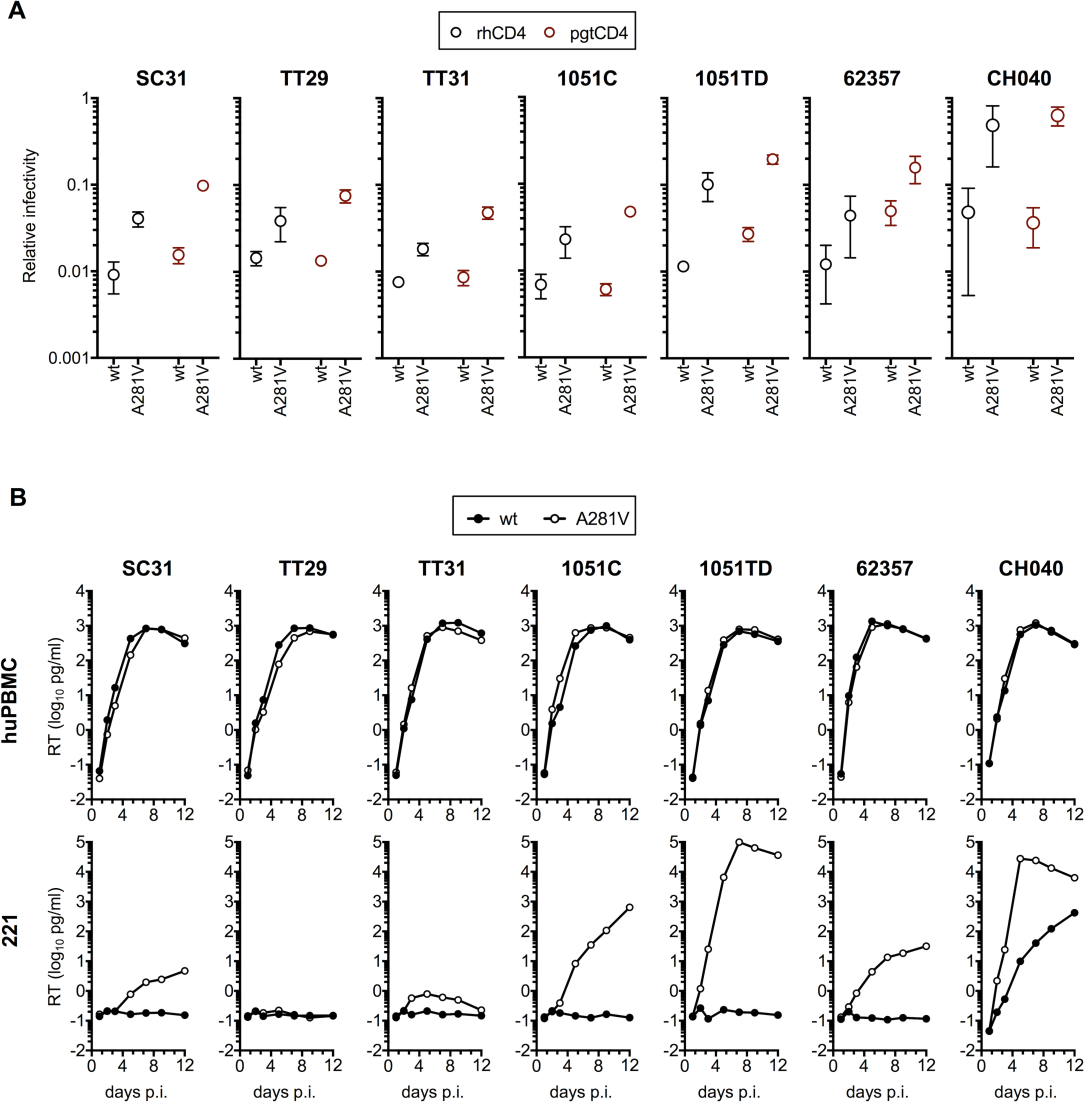
Role of aa 281 in macCD4 use of divergent HIV-1 envelope proteins. **(A)** SHIV infectivity on cells expressing human or macaque CD4 variants. SHIVs expressing the indicated parental or A281T mutant HIV-1 Env proteins were titrated on indicator Helios cells expressing hu/rh/pgtCD4. Infectivity was quantified by measuring luciferase expression in infected cells as Relative Light Units (RLU) and plotted as the ratio of RLU obtained in rh/pgtCD4-Helios over RLU obtained in huCD4Helios. Average and standard deviation from two independent experiments is shown. (**B)** Replication of SHIVs expressing the parental or mutants Env proteins indicated in huPBMC and rhesus 221 immortalized T cells. SHIV stocks were normalized for RT and 100pg RT of each virus was used per 1x10^5^ activated hu/rhPBMC. For 221 cells 50pg RT of each virus was used per 1x10^5^ cells. Replication was measured by RT in samples collected at the indicated times post-inoculation.

To determine whether A281 mutations could generally enhance the ability SHIVs to use macaque CD4, we introduced the A281V change into 7 randomly selected members of a library of SHIVs expressing T/F HIV-1 Env proteins [22]. Introduction of the A281V mutation did not affect envelope protein expression or incorporation into virions (S4A Fig). All the unmodified HIV-1 Env proteins exhibited poor infectivity on Helios cells expressing macaque CD4 variants compared to huCD4-Helios (Fig 6A), markedly lower than 1054 and AD8 Env proteins (Fig 5A and 5C). In all seven cases, the A281V change enhanced infectivity in cells expressing rh/pgtCD4, with effect sizes ranging from 2.5-fold to 10-fold for rhCD4 and 3-fold to 17-fold for pgtCD4 (Fig 6A). Moreover, the A281V change had dramatic consequences in the ability of SHIVs to replicate in macaque cells. All SHIVs replicated with comparable efficiency in human cells, however, with one exception (SHIV_CH040_) none replicated in macaque 221 cells (Fig 6B). Introduction of the A281V mutation did not affect replication in human cells but it conferred the ability to replicate in macaque 221 cells in most SHIVs (Fig 6B). In four of those SHIVs, SHIV_1051C_, SHIV_1051TD_, SHIV_62357_ and SHIV_CH040_, the effects were striking. In the remaining 2 SHIVs the A281V change resulted in only marginal improvements (SHIV_TT31_) or failed to improve replication efficiency in 221 cells (SHIV_TT29_) (Fig 6B). Interestingly, in these two SHIVs, the A281V mutation had the smallest effect on their ability to use rhCD4 (Fig 6A). Additionally, only the two SHIVS, SHIV_1051TD_ and SHIV_CH040_, for which the A281V substitution had the biggest effects on replication in 221 gained the ability to replicate in rhPBMC (S4B Fig). These results suggest that HIV-1 Env proteins are required to achieve a certain threshold ability to use macaque CD4 in order to replicate robustly in macaque cells and demonstrate an important role for residue 281 in expanding the tropism of HIV-1 Env proteins to include macaque CD4.

### Effects of A281 mutations on HIV-1 Env protein neutralization phenotypes

Although optimal macaque CD4 use is critical for efficient SHIV replication *in vivo*, for utility in their intended applications, it is also important that SHIV neutralization properties reflect those of parental HIV-1 strains circulating in humans [42]. Therefore, we determined the effects of the A281T/V mutation on SHIV neutralization tier classification (a representation of overall neutralization sensitivity) [43] and sensitivity/resistance to individual antibodies.

**Fig 7:**
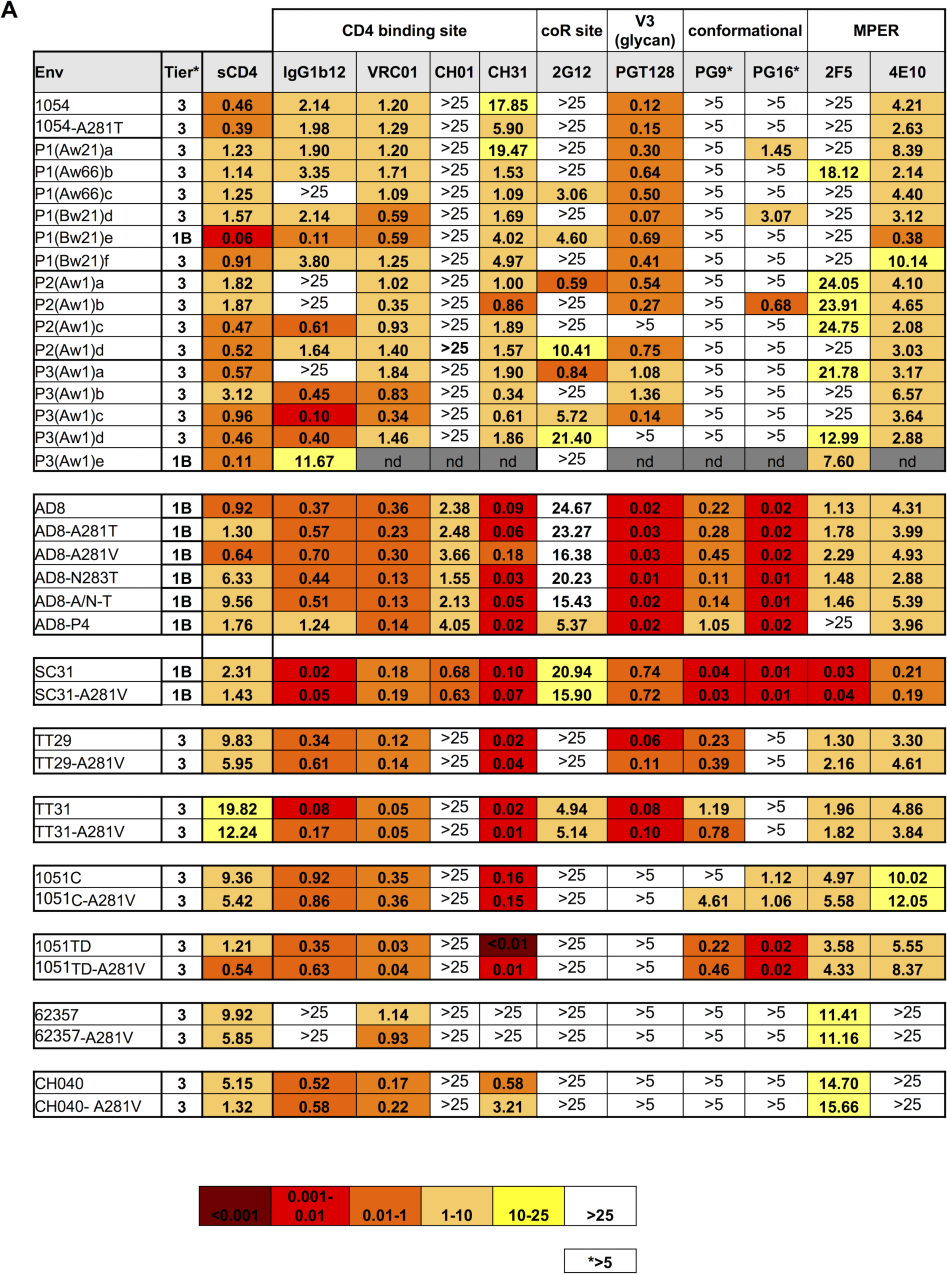
Env protein neutralization profile. Neutralization sensitivity of parental and mutant HIV-1 Env proteins used in this study. Neutralization tier phenotype was determined as previously described [43]. Neutralization sensitivity/resistance to soluble huCD4 (sCD4) and antibodies targeting the epitopes indicated is shown as the concentration (μg/ml) at which relative luminescence units were reduced 50% compared to virus control wells. For PG9 and PG16, 5μg/ml was the highest antibody concentration tested.

The introduction of the A281T mutation in 1054 did not alter the neutralization tier (S5 Fig) nor the sensitivity to soluble CD4 or the majority of antibodies tested (Fig 7). As might be expected, neutralization sensitivity to individual antibodies was altered for some of the adapted 1054 clones (Fig 7), that could potentially reflect selection attributable to particular immune responses in the animal from which they were derived. Furthermore, two clones P1(Bw21)e and P3(Bw1)e had acquired a substantially more neutralization sensitive tier classification compared to the parental Env protein (Fig 7 and S5 Fig). However, the majority of adapted clones maintained the neutralization resistant tier 3 phenotype of SHIV_1054_ (Fig 7 and S5 Fig).

Introduction of individual mutations A281T/V, N283T and A281T/N283T in SHIV_AD8_ did not substantially affect overall Env neutralization properties (Fig 7 and S5 Fig). SHIV_AD8-P4_ sensitivity to certain antibodies, such as IgGb12, 2G12 and 2F5 was different from the parental SHIV_AD8_ (Fig 7), a finding that is not unexpected given that this Env clone was derived following extensive *in vivo* adaptation. Nevertheless, this Env maintained the parental AD8 neutralization resistant tier 1B phenotype (S5 Fig) and bnAb neutralization properties (Fig 7).

Similarly, the additional seven SHIVs maintained their resistant phenotype upon introduction of the A281V mutations (Fig 7 and S5 Fig). Importantly, the introduction of mutation(s) at position 281 had little or no effect on the sensitivity of HIV-1 Env proteins AD8, SC31, TT29 TT31, 1051C and 1051TD to PG9 and/or PG16, antibodies that target conformational epitopes (Fig 7). This finding further supports the notion that A281V/T mutations do not affect overall Env conformation and thus do not alter Env sensitivity to various antibodies nor the overall neutralization phenotype.

## Discussion

Here, we demonstrate that a single aa, 281, in the CD4 binding site of HIV-1 Env is a determinant of the narrow tropism exhibited by HIV-1. We demonstrate that *in vivo* adaptation in macaques commonly results in a mutation at aa 281 that enhances the ability of HIV-1 Env to use macaque CD4, thereby improving replication in macaque cells without changing overall Env conformation or neutralization properties.

**Fig 8:**
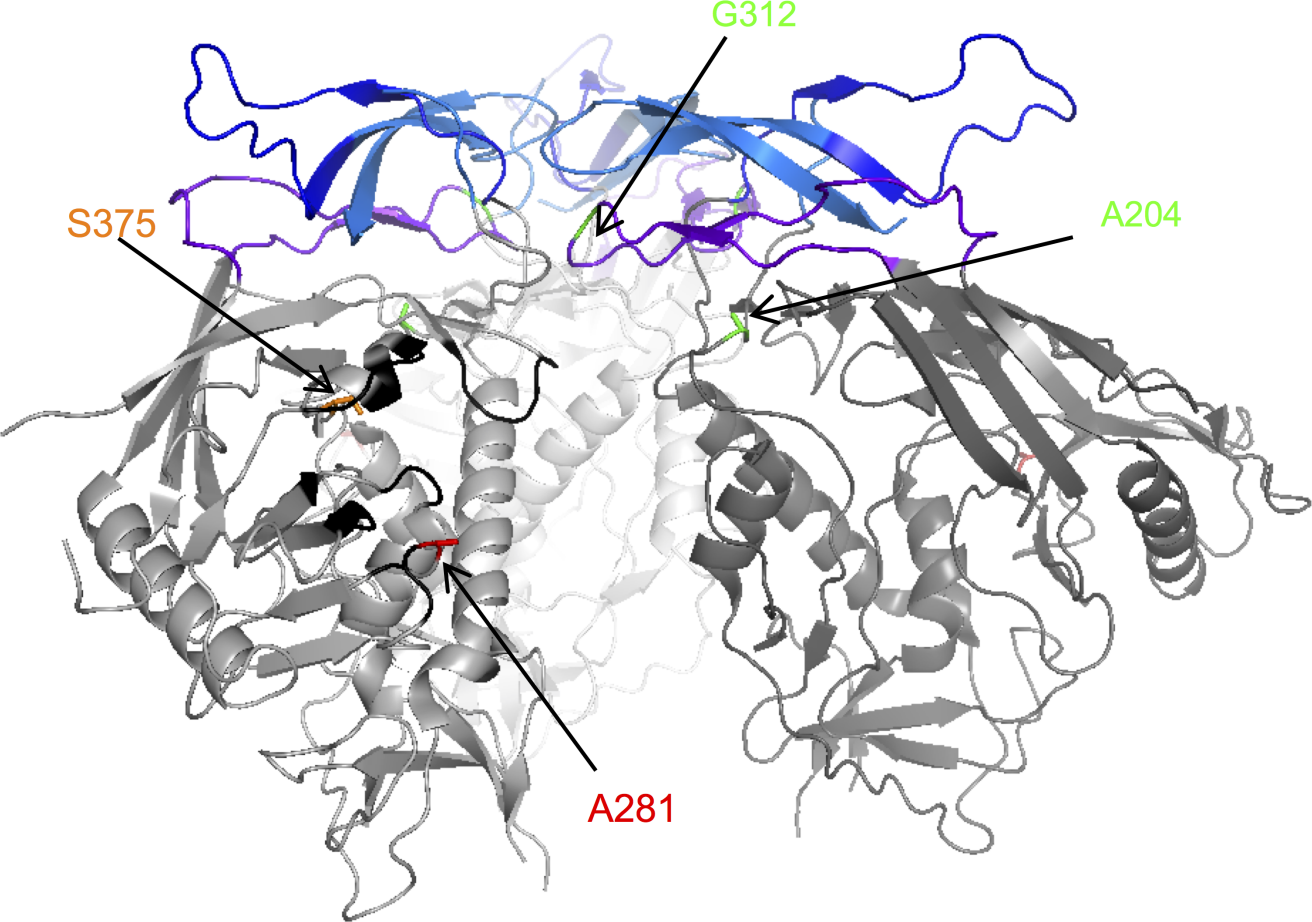
Env protein structure. Structure of gp120 trimer (modified from [44] using PyMol). Each gp120 monomer is shown in ribbon representation in a different shade of grey with the V1 and V2 loops in blue and the V3 loop in purple. The CD4-binding site is marked in black on the left monomer. Residue A281 is shown in red, residue S375 in orange and residues A204 and G312 in green and the side chains are depicted (lines).

Previous studies have reported that other individual HIV-1 Env residues can affect macaque CD4 use. Specifically, two aa changes, A204E and G312V, acquired during adaptation of a clade A SHIV in pigtail macaque cells *in vitro* significantly enhanced macaque CD4 use [27, 28]. These mutations also cause resistance to PG9 and PG16, antibodies known to recognize quaternary gp120 epitopes, while enhancing sensitivity to other antibodies. This finding suggests that A204E and G312V induce the adoption of a more ‘open’ Env protein conformation and increase both interaction with CD4 and overall neutralization sensitivity. Indeed, these residues are located distal to the CD4 binding site in the structure of the gp120 trimer [44] (Fig 8). It is possible that A204E/G312V changes arose *in vitro* because they were selected in the absence of immune pressure that might otherwise constrain Env proteins to neutralization resistant conformations. Interestingly, we did not observe changes at positions 204 or 312 in the SHIV sequences identified during *in vivo* adaptation, but did find G312 in about 30% of stHIV_AD8_ Env clones we obtained at necropsy from the P4 animal that succumbed to AIDS [25]. However, G312V was lost in subsequent stHIV_AD8_ passages and was not present in the AD8-P4 Env clone used in these studies.

Crucially, the *in vivo* adapted 1054 and AD8 Env proteins maintained the overall neutralization properties of the parental protein, with some exceptions (Fig 7). In contrast to results from *in vitro* selection experiments, we did not observe correlation between resistance to antibodies recognizing quaternary epitopes or acquisition of a neutralization sensitive phenotype (indicating adoption of a more ‘open’ conformation) with improved macaque CD4 use. Thus, HIV-1 Env proteins can apparently adapt to macaque CD4 in multiple ways with variable consequences, and it is clearly possible to derive viruses that can use macaque CD4 effectively yet maintain authentic neutralization properties.

The atypical inherent ability of some unadapted HIV-1 Env proteins to use macaque CD4, exemplified in 1054 and AD8, may underlie their comparative success in macaques and may be an important factor in determining why viruses expressing 1054 or AD8 Env proteins were selected during competition experiments *in vivo* [22, 45]. While, in contrast to 1054 and AD8, most HIV-1 Env proteins cannot use macaque CD4 efficiently, introduction of a single aa change, A281V, substantially improved replication in macaque cells in 5 out of 7 divergent clade B HIV-1 Env proteins (Fig 6). Residue 281 has previously been implicated in influencing HIV-1 Env protein neutralization sensitivity/resistance [46]. However, our data suggest that A281T/V directly affects the ability of HIV-1 Env proteins to specifically use macaque CD4 (Figs 5A and 6A) without large effects on neutralization sensitivity (Fig 7). It is also evident, that encoding T/V at position 281 is not always sufficient for improving replication in macaque cells. For example, the replication of SHIV_TT31_ and SHIV_TT29_ was not dramatically improved by the A281V mutation (Fig 6B). Of note, the A281V mutation was obtained during *in vitro* passage of the lab adapted NL4.3 Envelope protein in cells expressing common marmoset CD4 and CXCR4 and shown to be associated with increased binding of gp120 to marmoset CD4 that, like macaque CD4, has I at position 39 [47].

Recent studies have identified aa 375 as another determinant of macaque CD4 use in HIV-1 Env proteins [21]. Substitution of S375 with a mixture of the amino acids M/T/H/Y/W in four divergent Env proteins results in improved macaque CD4 usage and replication in macaque cells *in vitro* and in macaques *in vivo*. The preferred 375 residue selected following *in vivo* replication of mutant pools of SHIVs varied depending on the HIV-1 Env used [21]. Although residue 375 is not in the classical CD4 binding site [37], a long aa side chain at this position has the potential to affect interactions with Phe 43 on CD4 [21] (Fig 8). Of note, and in striking contrast to the consistent selection of A281 Env mutations upon serial passage *in vivo*, we have not observed mutations at position 375 in any of our *in vivo* passaged envelope proteins nor have mutations at this position been reported in serial passage experiments by other labs, despite the clear *in vivo* replicative advantages conferred by *in vitro* engineered mutations at this site. It will be interesting to determine the combined effects of A281 and S375 mutations.

Our data also demonstrate the impact of other HIV-1 Env residues on use of macaque CD4. Specifically, a single amino acid mutation, N283T, abolished the ability of SHIV_AD8_ to infect macaque CD4-expressing cells and replicate in macaque cells without affecting replication in human PBMC (Fig 5C and 5D). N283T is a key determinant of macrophage tropism in the AD8 Env [41]. Therefore, these data suggest a link between the ability of an Env protein to use low levels of CD4 (e.g. huCD4 on macrophages) and the ability to engage non-optimal CD4 variants (macaque CD4 on indicator/macaque T-cells). They also highlight the complexity of interactions between Env and CD4 that are dependent upon multiple residues making optimal contacts between the two proteins. Indeed, a recent study has revealed that a single CD4 molecule engages two gp120 monomers in the HIV-1 Env trimer through a novel CD4 binding site (CD4-BS2) on one of the monomers. This second binding site involves interactions between gp120 residues E/D62, E64, H66 and K207 and CD4 residues K22 and D63 and to a lesser extent K21 [48]. The gp120 CD4-BS2 site is highly conserved between HIV-1 group M Env proteins (and thus in all Env proteins used in this study) and we did not observe any changes therein during the *in vivo* adaptation of HIV-1 Env proteins in macaques (in our studies and other published sequences). Furthermore, CD4 residues involved in contacts with this second binding site in gp120 are conserved between human and macaque variants (Fig 3A). Even though adjacent CD4 residues S23 and I24 differ between human and macaque proteins, these differences do not affect the ability of macaque CD4 to act as a receptor for HIV-1 Env proteins [26]. Nevertheless, given the complexity of the gp120-CD4 interactions, it is rather remarkable that multiple divergent Env proteins have adopted the same solution to overcome their inability to use macaque CD4 efficiently through mutations at residue A281. Indeed, of all the changes described above, A281T/V is the only one to be consistently identified in divergent HIV-1 Env proteins adapted in macaques *in vivo*. In addition to the SHIV adaptations referenced in Fig 4, two clade C SHIVs, SHIV_1157i_ [49] and SHIV_CHN19_ [50] that replicate in rhesus and rhesus/pigtail macaques respectively, have Env proteins that encode a V at position 281. Finally, in a recent SHIV competition experiment with viruses expressing 7 divergent clade C Env proteins, the winner encoded T281 [19]. However, two SHIVs, SHIV_HxBc2P3.2_ [51] and SHIV_DH12_ [52] maintained A281 following adaptation in macaques suggesting that alternate infrequently used strategies to improve macaque CD4 use exist. Notably, both these SHIVs use CXCR4 rather than CCR5 and SHIV_HxBc2P3.2_ is based on the laboratory adapted HIV-1 HXB2c Env, that is highly neutralization sensitive and likely has an ‘open’ conformation, that should facilitate macaque CD4 use. Yet, in an independent adaptation experiment in rhesus and pigtail macaques SHIV_HxB_ acquired the A281T/V mutation in one animal [53].

We have previously demonstrated that overcoming species-specific restriction factors that determine HIV-1 host range is key to generating HIV-1 strains that can replicate in a new host [23, 25, 45]. It is evident that CD4 variation can present an additional barrier that Env proteins must negotiate in order for HIV-1 and SHIV strains to replicate optimally in macaques. We have identified a single aa that can contribute to a blueprint for manipulating HIV-1 Env proteins in a highly targeted way so that they can use macaque CD4 effectively. Such Env proteins should be better suited for the development of SHIVs that accurately represent HIV-1 strains circulating in humans as models for testing Env-directed prophylactic and therapeutic interventions against HIV-1.

## Materials and methods

### SHIV adaptation in rhesus macaques

Ten Indian origin rhesus macaques (Macaca mulatta; five females, five males, weighing 5–12 kg at study initiation) were used in accordance with protocols approved by the IACUC of the National Cancer Institute. For procedures requiring chemical immobilization and sedation, different anesthetics were used at the discretion of the attending veterinarian according to the IACUC approved protocol. For phlebotomy to obtain blood samples (within IACUC approved volume limits) anesthetics included ketamine (5–25 mg/kg, IM), telazol (4–10 mg/kg, IM), midazolam (200–500 μg/kg, IM), medetomidine (20–50 μg/kg, IM), and/or dexmedetomidine (7.5–15 μg/kg, IM). For euthanasia according to endpoints specified in the IACUC approved protocol, animals were initially sedated with ketamine (10–25 mg/kg, IM) or telazol (4–10 mg/kg, IM) followed by lethal overdose of sodium pentobarbital (>75 mg/kg, IV) to effect.

For serial passages from infected to naïve macaques, whole blood from donor animals was drawn into acid citrate dextrose (ACD) Vacutainer tubes (BD) immediately prior to i.v. infusion into the recipients (10ml per recipient animal). Plasma for viral RNA (vRNA) quantification and viral genomic sequence analysis and peripheral blood mononuclear cells (PBMCs) for flow cytometry assays were prepared from whole blood collected in EDTA anticoagulated Vacutainer tubes (BD). Plasma was separated from whole blood by centrifugation, recentrifuged to eliminate cells, platelets and debris, then aliquoted and then stored at -80°C until analysis. Plasma viral load determinations were performed as described previously [21].

### Single-genome amplification/sequencing of HIV-1 (SHIV) *env* and cloning into SHIV backbone

A limiting dilution, single-genome amplification PCR approach was used so that only one amplifiable molecule, encompassing the entire *env* gene, was present in each reaction. Reverse transcription of RNA to single-stranded cDNA was performed using SuperScript III reverse transcriptase (Invitrogen) according to manufacturer’s recommendations and a gene specific primer: SIVEnvR1 5’-TGT AAT AAA TCC CTT CCA GTC CCC CC-3’. The *env* gene was then amplified using a 1^st^ round PCR buffer supplemented with of 2 mM MgCl_2_, 0.2 mM of each deoxynucleoside triphosphate, 0.2 μM of each primer, and 0.025 U/μl Platinum *Taq* polymerase (Invitrogen) in a 20-μl reaction. First round PCR was performed with sense primer SIVEnvF1 5’-CCT CCC CCT CCA GGA CTA GC-3’ and antisense primer SIVEnvR1 under the following conditions: 1 cycle of 94°C for 2 min, 35 cycles at 94°C for 15 sec, 55°C for 30 sec, and 72°C for 4 min, followed by a final extension of 72°C for 10 min. Nested PCR was performed with primers SIVEnvF2 5’-TAT AAT AGA CAT GGA GAC ACC CTT GAG GGA GC-3’ and SIVEnvR2 5’-ATG AGA CAT RTC TAT TGC CAA TTT GTA-3’ under the same conditions used for first round PCR, but for a total of 45 cycles. Correctly sized amplicons were sequenced directly using inner PCR primers and 6 additional HIV-1 specific primers using Big Dye Terminator technology (Applied Biosystems). To confirm that PCR amplification was from a single template, chromatograms were manually examined for multiple peaks, indicative of the presence of amplicons resulting from PCR-generated recombination events, *Taq* polymerase errors or multiple variant templates in a single reaction.

Env coding sequences were amplified from selected SGA reactions using PCR and degenerate primers and introduced, using AgeI–XhoI, into a SHIV-KB9 plasmid backbone as previously described [22]. The sequence of some clones therefore differed from the SGA sequences in 1–5 amino acid positions generally clustering at the C-terminus (cytoplasmic tail) depicted in Fig 1C. Specifically, the following clones contained the indicated amino acid differences compared to their SGA counterparts: P1(Aw21)a-E186G/R456G/F721I/T723P, P1(Aw66)b-L721F, P1(Aw66)c-F721V/T723S, P1(Bw21)d-F721V/T723S, P1(Bw21)e- N448I/K500E/F721I/T723P, P1(Bw21)f-F210L/F721V/T723P, P2(Aw1)a-L721V/ T723P, P3(Aw1)a-N160K/F717L/F721I/T723P, P3(Aw1)b-S481R/ F721I/T723P, P3(Aw1)c-L721V/T723P, P3(Aw1)d-M86I/N553D/F721I/T723S, P3(Bw1)e-K683E/F717L/L721V/T723P/K734E.

### Macaque CD4 sequencing

To determine the coding sequences for CD4 proteins expressed by macaques, total RNA was extracted from 17 rhesus and 14 pigtail PBMC samples using TRIZOL (GibcoBRL) and cDNA synthesized using the SuperScript III RT kit (Invitrogen). CD4 coding sequences were amplified using primers based on an available macaque CD4 sequence (GenBank accession number D63347.1). Two or three independent PCR reactions were performed for each PBMC RNA sample and PCR products were introduced into the pCR-Blunt vector (Invitrogen) and analyzed by sequencing.

### Plasmid and cells

SHIV proviral plasmids encoding T/F HIV-1 Env proteins have been described previously [22]. Single amino acid changes in the HIV-1 Env coding sequences were introduced by overlap extension PCR. Adapted Env coding sequences were derived from plasma of SHIV-infected animals (see single-genome amplification methods). The SIV used in this study is SIV_MAC239_.

AD8 Env coding sequences were amplified by PCR from the unmodified HIV-1_AD8_ or an HIV-1 3’ genome half obtained from a P4-infected pigtail macaque using SGA [23].

All Env fragments were inserted using AgeI–XhoI into a SHIV-KB9 plasmid backbone following the same cloning strategy used to generate the parental T/F-SHIVs [22]. All virus stocks were produced by transient transfection of 293T cells using PEI (Polysciences).

Indicator HeLa cell lines, designated Helios, were generated using a self-inactivating retroviral vector that transduces an HIV-2 LTR-nanoluciferase-2A-GFP reporter cassette and a puromycin resistance gene. Following selection with puromycin single cell clones were derived by limiting dilution. A single cell clone with the highest difference in luciferase expression between infected and uninfected cells was selected and transduced with a retroviral vector expressing huCCR5 and hygromycin (LHCX, Clontech). Following selection with hygromycin and sorting of populations of cells with high levels of CCR5 expression, single cell clones were derived using limiting dilution and one such clone with uniform levels of CCR5 expression was selected for subsequent experiments (S2B Fig). huCD4, rhCD4 and pgtCD4 were introduced into Helios-R5 cells by retroviral gene transfer using LNCX2-based vectors (Clontech). Following G418 selection, cell populations expressing high levels of each CD4 variant were selected by sorting (S1C Fig).

Rhesus macaque 221 T-cells [29] were grown in RPMI medium with 20% FCS, 90 U/ml IL-2 (Peprotech) and gentamicin. Human peripheral blood mononuclear cells (PBMC) were isolated from leukopaks obtained from the New York Blood Center. Human and rhesus PBMC were isolated from blood by Ficoll-Paque gradient centrifugation. PBMC were activated with 5 μg/ml phytohemaglutinin (PHA-P; Sigma) and 20 U/ml IL-2 (Peprotech) for 48 h and then grown in the presence of 20 U/ml IL-2 in RPMI medium supplemented with 10% FCS. CD4^+^ T cells were enriched from rhPBMC by negative selection (CD4 T Cell Isolation Kit, Miltenyi). RhCD4^+^ T cells were activated with aCD2,/aCD3/aCD28 beads (T Cell Activation Kit, Miltenyi) per the manufacturer’s instructions for 72 hours and cultured with 100U/mL IL-2 in RPMI medium supplemented with 10% FBS (RPMI Complete) at a density of 2-3x10^6^ cells/ml. After 72 hours, activation beads were removed and cells were maintained in RPMI Complete with 100U/mL IL-2 for the duration of the experiment. 293T and HeLa cells were obtained from American Type Culture Collection (ATCC).

### FACS staining

Cells, (Helios, 1x10^6^) detached with 0.5 mM EDTA in PBS or hu/rhPBMC (1x10^6^), were stained in 100μl FACS buffer (2% FCS in PBS) with anti-CD4 (clone OKT4) conjugated with Alexa700 (Biolegend, 1/100) and anti-CD195 (CCR5) conjugated with PE (BD Pharmingen 550632, 1/20) for 30 min on ice. Fluorescence was measured using a LSRII (Becton Dickenson) and data analyzed using the FlowJo Software.

For whole blood cell counts in samples from infected animals, antibodies and reagents were obtained from BDBiosciences, unless indicated otherwise, and data analysis was performed using FCS Express (De Novo Software). Absolute cell counting was performed on EDTA-anticoagulated whole blood, as previously described [54, 55], using the following immunophenotyping panel: CD45 fluorescein isothiocyanate (FITC) (DO58-1283), CD3 phycoerythrin (PE) (SP34-2), CD4 allophycocyanin (APC) (L200), CD14 APC-Cy7 (M5E2; BioLegend), CD8α PE-Cy7 (SK1), and CD20 Pacific Blue (2H7; Bio-Legend). The samples were lysed, and approximately 50,000 CD45+ CD3+ cells were acquired for each sample to calculate cell counts, using a BD FACSVerse flow cytometer equipped with a volumetric flow sensor.

### SHIV replication (RT) assays

Individual SHIV stocks produced in 293T were normalized for RT and 100pg RT of each virus was used per 1x10^5^ freshly activated hu/rhPBMC. For 221 cells 50pg RT of each virus was used per 1x10^5^ cells. 16h post infection, cells were washed and supernatants collected over 14 days. The amount of virus was quantified using a one-step SYBR Green I based PCR RT assay [56]. Briefly, 5μl cell culture supernatant was incubated with 5μl 2x lysis buffer (0.25% Triton X-100, 50mM KCl, 100mM TrisHCl pH7.4, 40% glycerol) for 10 min at RT. The lysate was diluted 10x in 1x Core buffer: 5 mM (NH_4_)_2_SO_4_, 20 mM KCl and 20 mM Tris–HCl pH 8.3. 10μl of the sample were then mixed with 10μl of 2x reaction buffer: 5mM (NH_4_)_2_SO_4_, 20mM KCl and 20mM Tris–Cl (pH8.3), 10mM MgCl_2_, 0.2mg/ml BSA, 1x dilution of SYBR Green I (Life technologies S-7563), 400μM dNTPs, 1μM forward primer (TCCTGCTCAACTTCCTGTCGAG), 1μM reverse primer (CACAGGTCAAACCTCCTAGGAATG), 0.0002U/ml MS2 RNA (USBiological Life Sciences). RT reaction conditions were: 42°C for 20 min, 95°C for 2 min, followed by 40 cycles of 95°C for 5 sec, 60°C for 5 sec, 72°C for 15 sec and 80°C for 10 seconds measured using the StepOnePlus Real time PCR system (Life technologies). Each replication assay was repeated between 4 to 10 times and a representative experiment is shown. The RT assay was also used to quantify RT levels in viral stocks.

Replications in purified rhesus CD4+ T cells were performed by infecting 1x10^6^ activated RhCD4^+^ T cells at a nominal MOI of 0.02 in a total volume of 1mL. Infections were spinoculated at 800xg for 2 hours at room temperature, then incubated at 37C for 2 hours. At the end of this incubation period, cultures were washed 3 times to remove excess virus. Culture supernatants were collected over 14–17 days. Viral p27 protein was quantified by ELISA (Advanced Bioscience Laboratories).

### CD4 utilization assays

To determine efficiency of use of human and macaque CD4, Helios cells were seeded at 7x10^3^ cells/well in 96-well plates the day prior to infection. 3-fold dilutions of each virus stock were used for inoculation. 48 hours post-infection the percentage of infected cells was quantified by measuring luciferase expression using the Nano-Glo Luciferase assay (Promega). Statistical significance was calculated using the Fisher’s exact test to compare the infectivity values (Relative Light Units from two independent experiments) on huCD4-Helios and rh/pgtCD4-Helios of SHIV_AD8_ and each of the SHIV_AD8_ mutants.

### Neutralization assays

Neutralizing titers against SHIVs were determined in TZM-bl cells, as previously described [57]. Neutralizing titers against SHIVs were determined in TZM-bl cells, as previously described [57]. Briefly, neutralizing antibody activity was measured in 96-well culture plates by using Tat-regulated luciferase (Luc) reporter gene expression to quantify reductions in virus infection in TZM-bl cells. TZM-bl cells were obtained from the NIH AIDS Research and Reference Reagent Program, as contributed by John Kappes and Xiaoyun Wu. Test samples were diluted over a range of 1:20 to 1:43740 in cell culture medium and pre-incubated with different SHIVs (~150,000 relative light unit equivalents) for 1 hr at 37°C before addition of cells. Following a 48 hr incubation, cells were lysed and Luc activity determined using a microtiter plate luminometer and BriteLite Plus Reagent (Perkin Elmer). Neutralization titers are the sample dilution (for serum) or antibody concentration (for sCD4, purified IgG preparations and monoclonal antibodies) at which relative luminescence units (RLU) were reduced by 50% compared to RLU in virus control wells after subtraction of background RLU in cell control wells. Serum samples were heat-inactivated at 56°C for 1 hr prior to assay.

### Immunoblots

293T cells seeded at 106 cell/well in 6-well plates were transfected with 3μg of the indicated SHIV expression plasmid. 48hrs post-transfection the supernatant was harvested, filtered and virions purified by ultracentrifugation through a 20% sucrose cushion. Lysed virions and cells were separated by electrophoresis on NuPage 4–12% Bis-Tris gels (Novex) and transferred onto nitrocellulose membranes (GE Healthcare) that were probed with anti-gp120 HIV-1 goat antibody (12-62605-1, American Research Products) and an anti-SIV capsid mouse antibody (55-2F12, NIH AIDS Reagents Program).

### Accession numbers

Genebank accesion numbers for the 1054 adapted Env coding sequences are MF775894-MF776025 and for and rh/pgt-CD4 coding sequences MF769792-MF769804. Previously reported macaque CD4 coding sequences are: M31134, D63346, D63347, X73325, X73326, NM_001042662.

### Ethics statement

Ten Indian origin rhesus macaques (Macaca mulatta; five females, five males, weighing 5–12 kg at study initiation) were housed at the National Institutes of Health and cared for in accordance with American Association for the Accreditation of Laboratory Animal Care (AAALAC) standards in an AAALAC-accredited facility (Animal Welfare Assurance number A3081-01). The study was conducted under protocols approved by the Institutional Animal Care and Use Committee of the National Cancer Institute (Protocols AVP-013 and AVP-040) and adhered to the standards of the NIH Guide for the Care and Use of Laboratory Animals (National Research Council. 2011. Guide for the care and use of laboratory animals, 8th ed. National Academies Press, Washington, DC). At the start of the study, all animals were free of cercopithecine herpesvirus 1, simian immunodeficiency virus (SIV), simian type-D retrovirus, and simian T lymphotropic virus type 1. Animals with Mamu- alleles B*08 and B*17 were excluded from these studies. Animals on AVP-013 were used only as naïve donors of PBMC for *in vitro* experiments and amenable animals were socially housed with oversight from facility behavioral management staff. AVP-040 had an IACUC-approved exemption from social housing based on scientific justification and were housed in adjacent individual primate cages allowing interactions but no direct contact. Primary enclosures consisted of stainless steel primate caging provided by a commercial vendor. Animal body weights and cage dimensions were regularly monitored. Overall dimensions of primary enclosures (floor area and height) met the specifications of The Guide for the Care and Use of Laboratory Animals, and the Animal Welfare Regulations (AWR's). Further, all primary enclosures were sanitized every 14 days at a minimum, in compliance with AWRs. Secondary enclosures (room level) met specifications. All animals were housed under controlled conditions of humidity, temperature and light (12-hour light/12-hour dark cycles). Animals were fed commercial monkey chow, twice daily, with supplemental fruit or other produce at least three times per week. Filtered water was available ad libitum. Animals were observed at least twice daily by trained personnel, including behavioral assessments. Environmental enrichment included provision of species appropriate manipulatives, and foraging opportunities, as well music and video watching opportunities multiple times per week.

Human PBMC were purified from anonymous leukopaks obtained from the New York Blood Center.

## Supporting information

S1 Fig CD4 T cell counts in infected animals and *in vitro* replication of SHIVs expressing adapted 1054 Env proteins. **(A)** CD4+ T cell counts in SHIV_1054_-infected macaques. Peripheral CD4+ T cells were measured in animals from P2 and P3 from Fig 1A using same color scheme: P2A dark orange, P2B red, P3A dark green, P3B light green. **(B)** Replication of SHIVs expressing the parental or *in vivo* adapted P3 clones of 1054 in purified rhesus CD4+ T cells. CD4^+^ T cells were enriched from rhPBMC by negative selection and activated. 1x10^6^ activated RhCD4^+^ T cells were infected with individual SHIVs at an MOI of 0.02 using spinoculation. Supernatants were collected over 14–17 days. Viral p27 protein was quantified by ELISA.
(TIFF)

S2 Fig Comparison of human and macaque CD4. **(A)** Polymorphisms in aa positions between rhesus CD4 variants and pigtail CD4 variants. Logo analysis of the probability of having each aa at the indicated position using two variants per animal obtained from 17 rhesus and 14 pigtail macaques. Numbering starts at the first residue of the D1 domain (aa 25 in the CDS). **(B)** Cell surface expression of CCR5 and each CD4 variant on Helios cells determined by FACS. Helios cells stably expressing the indicated receptors were stained with anti-CCR5 antibody conjugated to PE and anti-CD4 antibody conjugated to Alexa700 that recognizes both human and macaque proteins as demonstrated in (C). (**C)** Cell surface expression of CD4 on activated human and rhesus PBMC using the anti-CD4 antibody that recognizes both human and rhesus CD4 (used in (B)).
(TIFF)

S3 Fig Relative infectivity of SHIVs expressing adapted 1054 Envs to parental SHIV_1054_. Infectivity of SHIVs expressing the indicated Env proteins was measured in huCD4-Helios and is expressed as a ratio over the infectivity of the SHIV expressing the parental, unadapted 1054 Env. RT activity of SHIV stocks used was comparable within experiments. Average and standard deviation of two independent experiments is shown.
(TIFF)

S4 Fig Effects of aa 281 on divergent HIV-1 envelope proteins. **(A)** Env protein expression. Immunoblots of cellular and purified virion lysates from cells transiently transfected with SHIVs expressing the indicated HIV-1 Env unmodified or with the A281V mutation. Blots were probed with antibodies recognizing the HIV-1 Env (gp120) or SIV_MAC_ CA (p27) proteins. **(B)** Replication of SHIVs expressing the parental or mutants Env proteins indicated in rhPBMC. SHIV stocks were normalized for RT and 100pg RT of each virus was used per 1x10^5^ activated rhPBMC. Replication was measured by RT in samples collected at the indicated times post-inoculation.
(TIFF)

S5 Fig Neutralization of SHIVs by reference plasmas. Neutralization of the wild type and mutants SHIVs indicated using a panel of reference plasma samples in the TZM-bl assay system. Values are plasma dilution at which relative luminescence units (RLUs) were reduced compared to virus control wells. The geometric mean titers (GMT) were calculated for each virus and the range of GMT corresponding to each neutralization tier designation is shown.
(TIFF)
