# Supplementary figures and images for: A single gp120 residue can affect HIV-1 tropism in macaques
Source: PLoS Pathog. 2017 Sep 25;13(9):e1006572. doi: 10.1371/journal.ppat.1006572 (PMC5629034; doi:10.1371/journal.ppat.1006572)

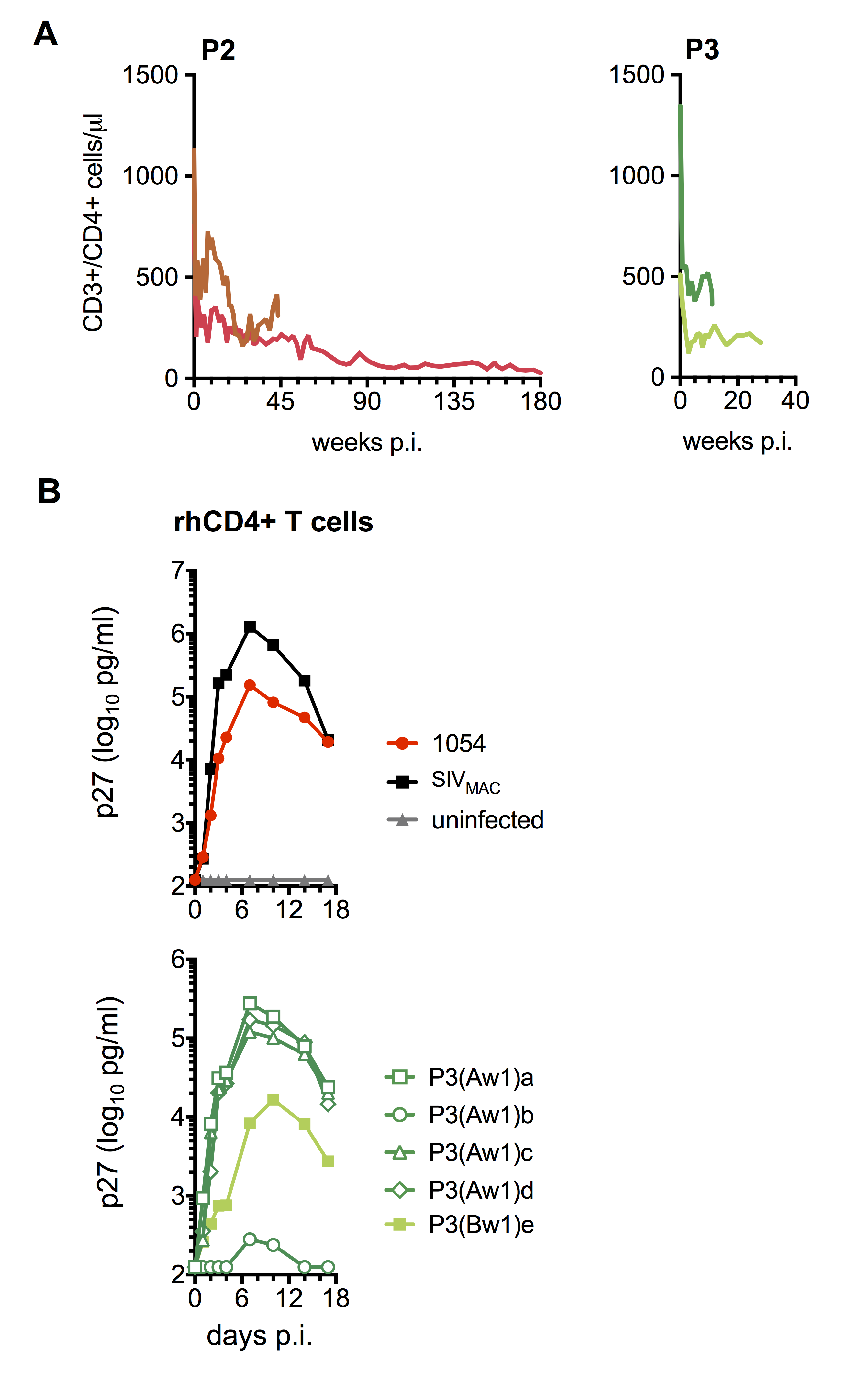

Supplement: S1 Fig — (A) CD4+ T cell counts in SHIV1054-infected macaques. Peripheral CD4+ T cells were measured in animals from P2 and P3 from Fig 1A using same color scheme: P2A dark orange, P2B red, P3A dark green, P3B light green. (B) Replication of SHIVs expressing the parental or in vivo adapted P3 clones of 1054 in purified rhesus CD4+ T cells. CD4+ T cells were enriched from rhPBMC by negative selection and activated. 1x10^6^ activated RhCD4+ T cells were infected with individual SHIVs at an MOI of 0.02 using spinoculation. Supernatants were collected over 14–17 days. Viral p27 protein was quantified by ELISA. (TIFF) [file ppat.1006572.s001.tiff]

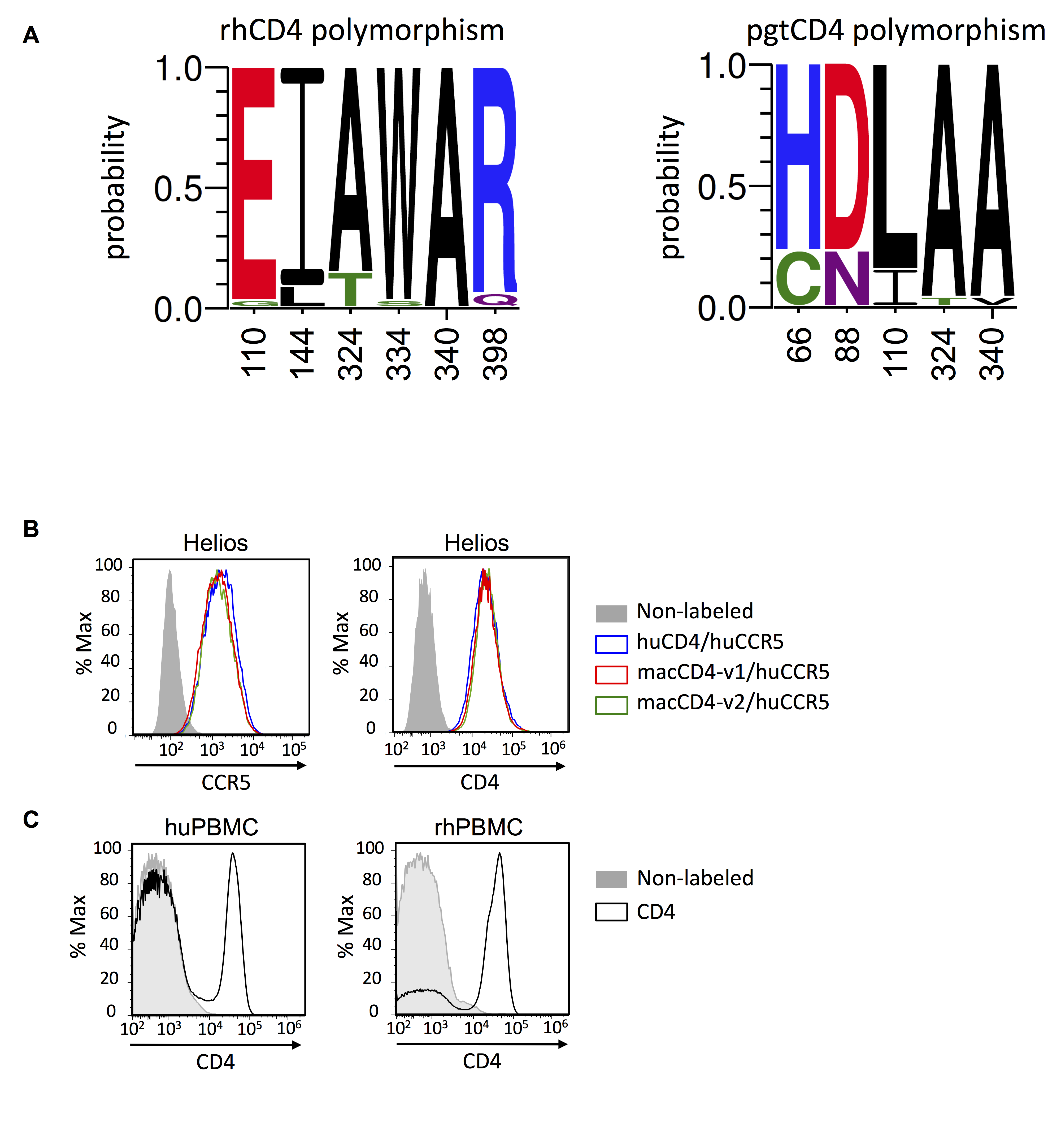

Supplement: S2 Fig — (A) Polymorphisms in aa positions between rhesus CD4 variants and pigtail CD4 variants. Logo analysis of the probability of having each aa at the indicated position using two variants per animal obtained from 17 rhesus and 14 pigtail macaques. Numbering starts at the first residue of the D1 domain (aa 25 in the CDS). (B) Cell surface expression of CCR5 and each CD4 variant on Helios cells determined by FACS. Helios cells stably expressing the indicated receptors were stained with anti-CCR5 antibody conjugated to PE and anti-CD4 antibody conjugated to Alexa700 that recognizes both human and macaque proteins as demonstrated in (C). (C) Cell surface expression of CD4 on activated human and rhesus PBMC using the anti-CD4 antibody that recognizes both human and rhesus CD4 (used in (B)). (TIFF) [file ppat.1006572.s002.tiff]

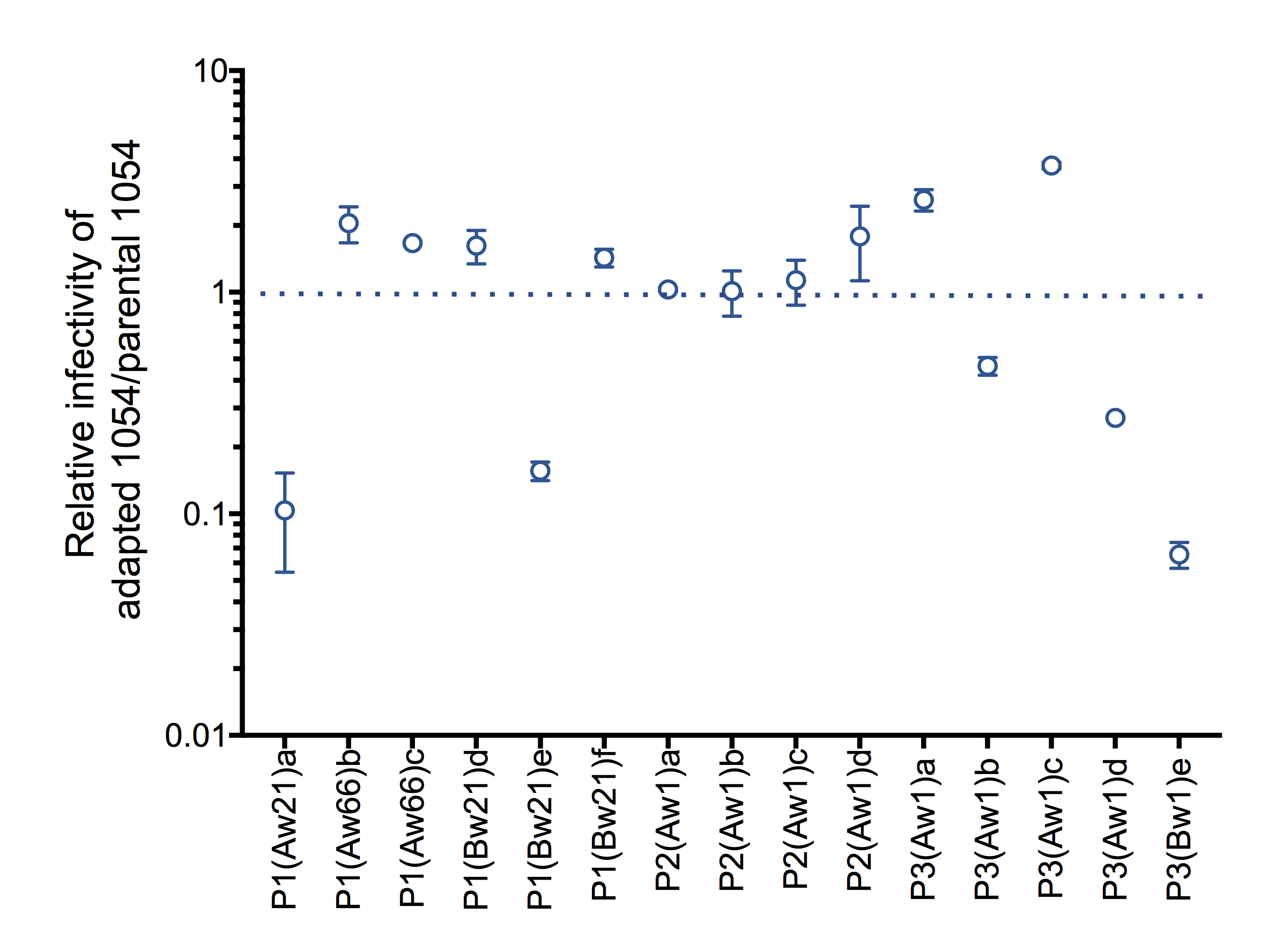

Supplement: S3 Fig — Infectivity of SHIVs expressing the indicated Env proteins was measured in huCD4-Helios and is expressed as a ratio over the infectivity of the SHIV expressing the parental, unadapted 1054 Env. RT activity of SHIV stocks used was comparable within experiments. Average and standard deviation of two independent experiments is shown. (TIFF) [file ppat.1006572.s003.tiff]

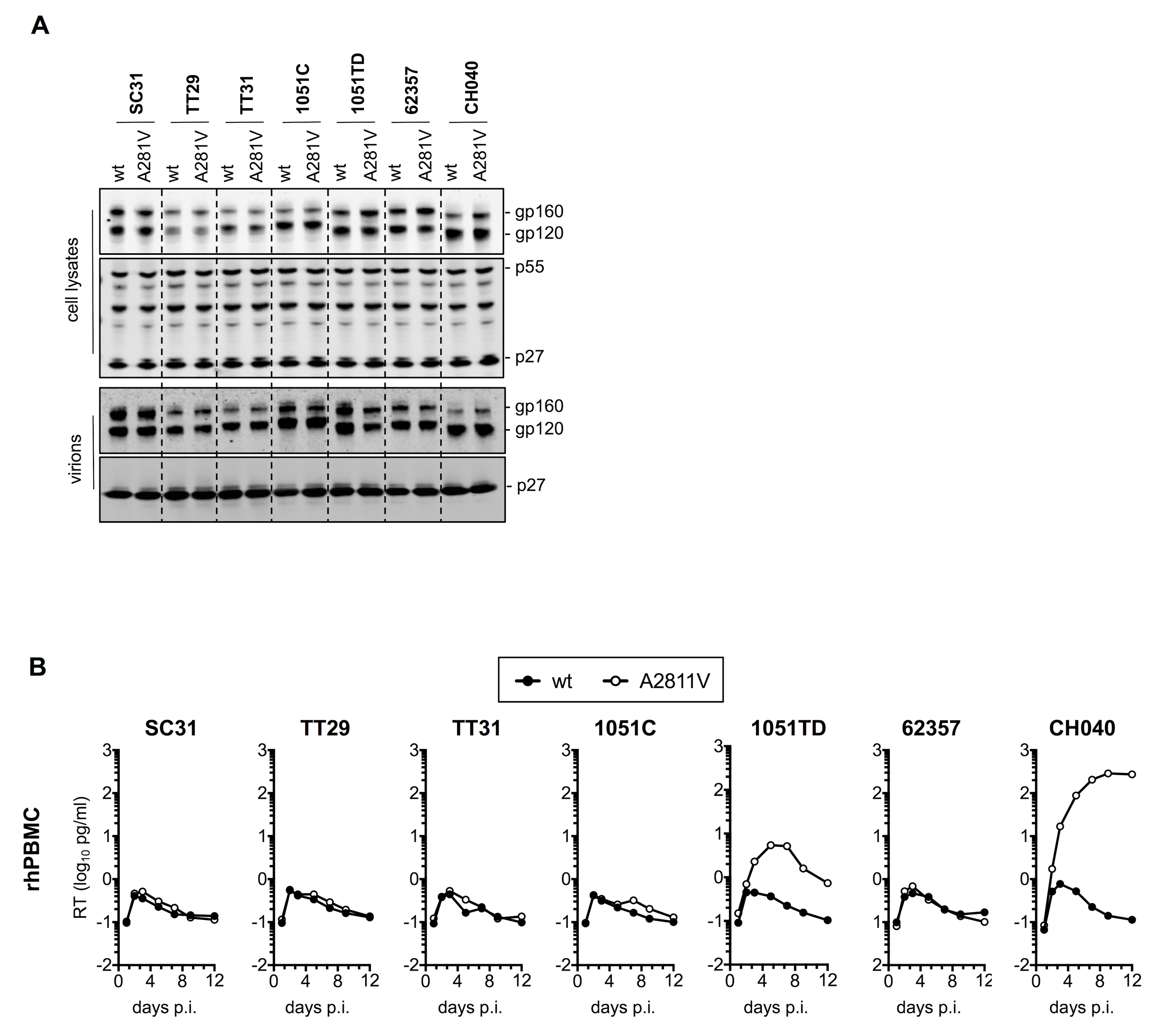

Supplement: S4 Fig — (A) Env protein expression. Immunoblots of cellular and purified virion lysates from cells transiently transfected with SHIVs expressing the indicated HIV-1 Env unmodified or with the A281V mutation. Blots were probed with antibodies recognizing the HIV-1 Env (gp120) or SIVMAC CA (p27) proteins. (B) Replication of SHIVs expressing the parental or mutants Env proteins indicated in rhPBMC. SHIV stocks were normalized for RT and 100pg RT of each virus was used per 1x10^5^ activated rhPBMC. Replication was measured by RT in samples collected at the indicated times post-inoculation. (TIFF) [file ppat.1006572.s004.tiff]

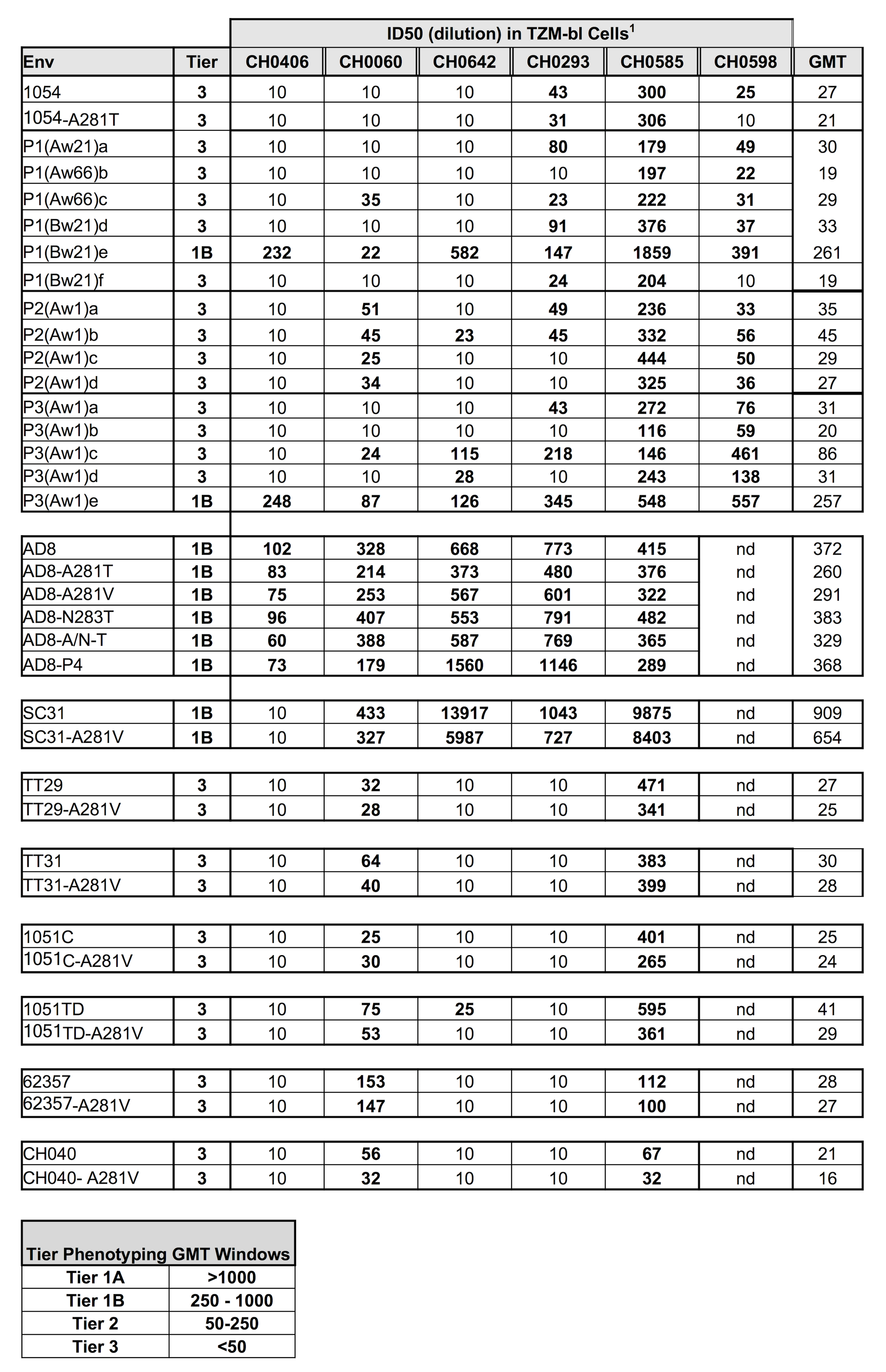

Supplement: S5 Fig — Neutralization of the wild type and mutants SHIVs indicated using a panel of reference plasma samples in the TZM-bl assay system. Values are plasma dilution at which relative luminescence units (RLUs) were reduced compared to virus control wells. The geometric mean titers (GMT) were calculated for each virus and the range of GMT corresponding to each neutralization tier designation is shown. (TIFF) [file ppat.1006572.s005.tiff]
